# Preliminary division of not socially parasitic Greek *Temnothorax* Mayr, 1861 (Hymenoptera, Formicidae) with a description of three new species

**DOI:** 10.3897/zookeys.877.36320

**Published:** 2019-10-02

**Authors:** Sebastian Salata, Lech Borowiec

**Affiliations:** 1 Department of Entomology, California Academy of Sciences, San Francisco, CA 94118, USA California Academy of Sciences San Francisco United States of America; 2 Department of Biodiversity and Evolutionary Taxonomy, University of Wrocław, Wrocław, Poland University of Wrocław Wrocław Poland

**Keywords:** Balkan Peninsula, *Temnothorax aveli* group, *Temnothorax nylanderi* group, taxonomy

## Abstract

The division of Greek members of the genus *Temnothorax* into 17 morphological groups is proposed. *Temnothorax aveli* species group is reviewed with three species: *T. turcicus* (Santschi) (North Aegean Islands, Sterea Ellas, Peloponnese and Thessaly), and two species new to science: *Temnothorax brackoi***sp. nov.** (Epirus, Ionian Islands, Macedonia, Peloponnese, western Sterea Ellas, Thessaly, and also Dalmatia in Croatia), and *T. messiniaensis***sp. nov.** (Ionian Islands and Peloponnese); a new species *Temnothorax triangularis***sp. nov.**, a member of the *Temnothorax nylanderi* species group is also described (Sterea Ellas: Euboea Island).

## Introduction

The Myrmicinae genus *Temnothorax* Mayr, 1861 is one of the most speciose in the Mediterranean Region (sensu Vigna Taglianti et al. 1999). Among 279 Palearctic taxa (Bolton 2019), 194 are known from this region (Borowiec 2014, Radchenko et al. 2015, Salata and Borowiec 2015, Csősz et al. 2015, 2018, Galkowski and Lebas 2016, Galkowski and Cagniant 2017, Sharaf et al. 2017, Catarineu et al. 2017, Salata et al. 2018). Most of the recent research performed on this genus was focused on taxa from European and Anatolian parts of the Mediterranean Region. Those areas are considered as most diverse and species-rich, and each further study confirms this assumption. Greece, located at the joint of European and Anatolian subunits, hosts numerous and various *Temnothorax* taxa and so it is one of the most challenging fields of research. Study on Greek *Temnothorax* is additionally impeded by a complicated taxonomical history of several taxa and a lack of new material. Moreover, recent studies published by Csősz et al. (2015, 2018) revealed that species considered as a widespread represent in fact complexes of cryptic taxa.

As most of *Temnothorax* species are morphologically variable, to determine their distribution range and morphological variability, researchers ought to investigate rich material. During several field trips to various places on Greek islands and the mainland, we collected more than 1000 samples of *Temnothorax* species from 480 localities. The material collected during those expeditions helped us to establish morphological variability within species and divide collected material into several species groups. In most cases, a division into species group is based on species morphology only, and we did not verify if it corresponded with the phylogeny of this genus. This method is a widely accepted tool helping in determining the range of studied material and is commonly practiced (Csősz et al. 2018, Salata and Borowiec 2015, Galkowski and Cagniant 2017, Catarineu et al. 2017). However, we adjusted our species-group definitions to phylogenetical data provided by Prebus (2017) and Csősz et al. (2015).

Some Greek *Temnothorax* have been already included in studies on species groups of *T. nylanderi* (Csősz et al. 2015), *T. interruptus* (Csősz et al. 2018), *T. recedens* (Salata and Borowiec 2015), and *T. muellerianus* (Salata and Borowiec 2015). Below we present our division of *Temnothorax* from Greece, which will be used as a base for further studies and we describe three new species: one of them, *Temnothorax triangularis*, is a member of recently revised *T. nylanderi* species group (Csősz et al. 2015). Two others are a widespread Greek species, members of the *Temnothorax aveli* species group. Because *Temnothorax* taxa known from Crete were revised recently (Salata et al. 2018), and the majority of its endemic species creates single-species groups unknown from other Greek regions, they were excluded from this paper. Also, socially parasitic members of *Temnothorax*, often classified in separate genera *Chalepoxenus* Menozzi and *Myrmoxenus* Ruzsky, are not included in this study (see discussion in Seifert et al. 2016 and Ward et al. 2016).

## Materials and methods

Most of the material was sampled between 2007 and 2019 from sites in different parts of Greece. The main method was direct sampling (hand collecting). Individual specimens and nests were collected on the ground, in leaf litter and rock rubble, under stones, on tree trunks, and in dry twigs of herbs. This method was occasionally supplemented by litter sifting and collecting material with an entomological umbrella. All specimens were preserved in 75% EtOH. Study was supported with material deposited in the collection of G. Bračko (Ljubljana, Slovenia). All studied type specimens of taxa mentioned in differential diagnosis or characteristics are listed below.

Examined specimens are housed in the following collections:

**BMNH**Natural History Museum, London;

**CASC**California Academy of Sciences, San Francisco, California, USA;

**DBET** Department of Biodiversity and Evolutionary Taxonomy, University of Wrocław, Poland;

**MHNG**Museum d’Historie Naturelle, Geneve, Switzerland;

**HNHM**Hungarian National History Museum, Budapest, Hungary;

**NHMW**Natural History Museum, Vienna, Austria;

**MNHW** Museum of Natural History, University of Wrocław, Wrocław, Poland;

**MZLS**Museum of Zoology, Lausanne, Switzerland;

**NHMB**Naturhistorisches Museum Basel, Switzerland;

**PWC** Petr Werner collection, Prague, Czech Republic;

**SMNG**Senckenberg Museum für Naturkunde Görlitz, Görlitz, Germany;

**ZMHB**Museum für Naturkunde, Zentralin-stitut der Humboldt-Universität, Berlin, Germany.

To determine a distribution range and a morphological variability of the new species we compared them with material collected from other Greek regions. Data concerning samples used in the comparison is provided in series of regional checklists (Borowiec and Salata 2012, 2013, 2017a, b, 2018a, b, c, d, e, Bračko et al. 2016) and we see no reason to repeat this information.

Specimens were compared using standard methods of comparative morphology. All measurements were made in μm using a pin-holding stage, permitting rotations around X, Y, and Z axes. A Nikon SMZ18 stereomicroscope was used at a magnification of ×100 for each character. Photographs were taken using a Nikon SMZ 1500 stereomicroscope, Nikon D5200 camera and Helicon Focus software. All given label data of type specimens are in original spelling, presented in square brackets; a vertical bar (|) separates data on different rows and double vertical bars (||) separate labels. Images of type specimens are available online on AntWeb (www.AntWeb.org) and are accessible using the unique CASENT or FOCOL identifying specimen code. If not stated differently material deposited in Museum of Natural History, University of Wrocław (in permanent deposit in the Department of Biodiversity and Evolutionary Taxonomy).

Pilosity inclination degree applies to this used in Hölldobler and Wilson (1990). The adpressed (0–5°) hairs run parallel, or nearly parallel to the body surface. Decumbent hairs stand 10–15°, subdecumbent hair stands 30°, suberect hairs stand 35–45°, and the erect hairs stand more than 45° from the body surface.

Measurements:

**EL** eye length; measured along the maximum vertical diameter of eye;

**EW** eye width; measured along the maximum horizontal diameter of eye;

**HL** head length; measured in straight line from mid-point of anterior clypeal margin to mid-point of posterior margin in full-face view;

**HW** head width; measured in full-face view directly above the eyes;

**PEH** petiole height; measured in lateral view, the chord of ventral petiolar profile at node level is the reference line perpendicular to which the maximum height of petiole is measured;

**PEL** petiole length; measured in lateral view, from anterior corner of subpetiolar process to dorsocaudal corner of caudal cylinder;

**PNW** pronotum width; maximum width of pronotum in dorsal view;

**PPH** postpetiole height; measured perpendicularly to a line defined by the linear section of the segment border between dorsal and ventral petiolar sclerite;

**PPL** postpetiole length; maximum length of the postpetiole measured in lateral view perpendicular to the straight section of lateral postpetiolar margin;

**PPW** postpetiole width; maximum width of postpetiole in dorsal view;

**PSL** propodeal spine length; measured from the centre of the propodeal spiracle to the top of the propodeal spine in lateral view;

**PW** petiole width; maximum width of petiole in dorsal view;

**SDL** spiracle to declivity length; minimum distance from the centre of the propodeal spiracle to the propodeal declivity;

**SL** scape length; maximum straight-line length of scape excluding the articular condyle;

**WL** mesosoma length; measured as diagonal length from the anterior end of the neck shield to the posterior margin of the propodeal lobe.

Indices:

**CI**HW/HL * 100;

**EI1**EW/EL * 100;

**EI2**EW/HL * 100;

**SI1**SL/HL * 100;

**SI2**SL/HW * 100;

**MI**PNW/WL * 100;

**PI** PL/PH * 100;

**PPI**PPL/PPH * 100;

**PSI**PSL/SDL * 100.

Abbreviations:

**g.** gyne;

**m.** male;

**w.** worker.

We decided to list all other ant species collected from the same localities as species new to science. In our opinion, it provides valuable information about ecosystem structure and species diversity characteristic for habitats preferred by these species.

### Type material of species noted in the comparative diagnoses

*Temnothorax affinis* (Mayr, 1855), syntype (w.): Oesterreich || Type || *Leptothorax* | *affinis* | Mayr || 29889 || *affinis* | Mayr || GBIF-D/FFoCol | 2006 (ZMHB); syntype (w.): Oesterreich || Type || *Leptothorax* | *affinis* | Mayr || GBIF-D/FoCol | 2007 (ZMBH), available from photo in AntWeb (FOCOL2006);

*Temnothorax aveli* (Bondroit, 1918), syntype (w.): Lept. aveli | Bondr. || Type || *Leptothorax* | *Aveli* Bondr. | type – Sayat || Sammlung | Dr. F. Santschi | Kairouan || ANTWEB | CASENT0912907 (NHMB), available from photo in AntWeb (CASENT0912907);

*Temnothorax artvinensis* Seifert, 2006, holotype (w.): ARTVIN – 5km SW Artvin | 100 Mh 1164 | Kiefernwald 50% | Leg. Schulz 27.06.93 TURKEI || Holotype | *Temnothorax* | *artvinense* | SEIFERT || GBIF-D/FoCol | 1361 (SMNG), available from photo in AntWeb (FOCOL1361);

*Temnothorax graecus* (Forel, 1911), lectotype (w.): L. bulgaricus | For. | r. graecus | type Forel | Patras … | … (Forel) || Lectotype | *Leptothorax graecus* | Forel, 1911 top specimen | det. A. Schulz & M. Verhaagh 1999 || Typus || r. d. graecus | Forel || Coll. | A. Forel || ANTWEB | CASENT0909017 (MHNG), examined; paralectotype (w.): the same pin as lectotype, bottom specimen (MHNG), examined; paralectotypes (2w.): Typus || L. bulgaricus | For. | r. graecus | Forel | type | Amaroussia | p. Athenes (Forel) || coll. | A. Forel (MHNG), examined.

*Temnothorax kemali* (Santschi, 1934), syntype (w.): *Leptothorax* | *kemali* Sant | Type || Izmir | 29.VII.33 | Santschi || Type || *Leptothorax* | *kemali* | A. Schulz det || Basel 10 || ANTWEB | CASENT0912952 (NHMB), available from photo in AntWeb (CASENT0912952);

*Temnothorax laconicus* Csősz, Seifert, Müller, Trindl, Schulz & Heinze, 2015, paratype (w.): *Temnothorax laconicus* || *Temnothorax laconicus* | Csosz et al. 2014 | PARATYPE || PARATYPE || GREECE, GRE 2011-0345 | Taygethos Oros, Street to Profiti | Ilias | 36.968N, ; 22.404E, ; 800 m | leg. A. Schulz, 01.05.2011 || ANTWEB | CASENT0914696 (HNHM), available from photo in AntWeb (CASENT0914696);

*Temnothorax lagrecai* (Baroni Urbani, 1964), paratype (w.): Caltagirone | Sichel leg. || BOSCO S. PIETRE | 15.IV.62 || TYPUS || ANTWEB | CASENT0919741 (NHMW), available from photo in AntWeb (CASENT0919741)

*Temnothorax nigriceps* (Mayr, 1855), syntype (w.): *L. tubero* | *nigriceps* | Mt. Tendre | … || Typus || Coll. | A. Forel ||ANTWEB | CASENT0909038 (MHNG), examined;

*Temnothorax rabaudi* (Bondroit, 1918), paralectotype (w.): *Leptothorax* | *Rabaudi* | Boundr. | type | Gironde || ANTWEB | CASENT0904752 (MSNG), available from photo in AntWeb (CASENT0904752)

*Temnothorax sordidulus* (Müller, 1923), syntype (w.): No: 175 Types | *L. carinthiacus* | Bernard || TYPE || 20.4.56 | … || Carinthia | Viktring | Holzel leg. || ANTWEB | CASENT0907632 (MZLS), available from photo in AntWeb (CASENT0907632);

*Temnothorax tauricus* (Ruzsky, 1902), cotypes, (3w.): L. unifasciatus | … | r. tauricus | Ruzsky | Crimee || Cotypus || v. L. *tauricus* | Ruzsky || 1288 || Coll. Forel || ANTWEB | CASENT0909049 (MHNG), examined; cotypes (3w.): L. unifasciatus | Latr. | tauricus | Ruzsky | Crimee || 1288 || cotype (MHNG), examined.

## Taxonomy

### Species groups of Greek *Temnothorax*

The number of *Temnothorax* taxa known from Greece is estimated at 59 (including undescribed species) and include recent data published in taxonomic revisions and faunistic papers (Salata and Borowiec 2018). Below we present series of morphological characters that were used in species-groups division. Some of the species groups were defined in former publications and we used those descriptions as a base for our research. This applies to the following cases: *bulgaricus* species group (Radchenko 1995a); *affinis* species group, *tuberum* species group, *corticalis* species group, and *clypeatus* species group (Radchenko 1995b); *nylanderi* species group (Radchenko 1995c, Csősz et al. 2015); *exilis* species group (Cagniant and Espadaler 1997); *interruptus* species group (Csősz et al. 2018); and *recedens* species group (Salata and Borowiec 2015). However, in some cases we adapted those definitions to Greek species and thus they differ from the original definitions. We also list members of each species group known from Greece. Taxa known and described from Crete are not included in this division, as they were recently revised in a separate paper (Salata et al. 2018), and most of them create separate, single species groups, so far known only from this island.

*Temnothorax affinis* species group: antennae 12-segmented, club not or only slightly darkened, metanotal groove absent, body colouration orange to dark orange with darker head and dark first gastral tergite, propodeal spines very long and thin, straight or only slightly curved, petiole node subangular in profile, head and mesosoma surface moderately sculptured, head completely microsculptured and with more or less developed longitudinal ridges. In Greece only one arboreal species, *T. affinis* (Mayr).

*Temnothorax angustulus* species group: antennae 12-segmented, club darkened, metanotal groove feebly marked, body colouration mostly brown to almost black, including head, first gastral tergite mostly brown with yellowish brown basal spot, propodeal spines short to long but thin, straight or slightly curved, petiole node angulate in profile, head and mesosoma surface finely sculptured, head only partly microsculptured, central part more or less shiny, longitudinal ridges diffused visible only in ocular area and sides of head. In Greece only one species known from Mediterranean coniferous forests, *T. dessyi* (Menozzi).

*Temnothorax anodontoides* species group: antennae 12-segmented, club darkened, metanotal groove absent, body colouration brown, dark brown to almost black including head, first gastral tergite completely brown to almost black not or only slightly paler at base, propodeal spines very short with broad base, not longer than basal width, petiole node rounded in profile, head and mesosoma surface very strongly sculptured appear partly rugose, head background microsculptured, whole surface with longitudinal ridges or rugosities. In Greece at least three undescribed species known only from high montane localities.

*Temnothorax aveli* species group: antennae 12-segmented, completely yellow but club sometimes slightly darkened, metanotal groove absent, body colouration mostly yellow including head, sometimes only gena and femora slightly darkened, first gastral tergite with narrow to broad dark posterior band, always with paler spot at base, propodeal spines short to moderately long, with broad base, petiole node rounded in profile, head and mesosoma surface finely sculptured, head almost uniformly, regularly microsculptured, mostly without longitudinal ridges, sometimes only central part of head with narrow area of diffused microreticulation, but head never appears smooth and shiny – in Greece three, revised below, species mostly known from lowland habitats, especially Mediterranean forests and bushes, nestling inside dry stems of trees and twigs of bushes and large herbs– *T. brackoi* sp. nov., *T. messiniaensis* sp. nov., *T. turcicus* (Santschi, 1934), (?)*T. tauricus* (Ruzsky) (see comments in the redescription of *T. turcicus*).

Comments: Members of the *aveli* species group are morphologically similar to those assigned to the *tuberum* species group. Despite morphological similarities taxa of both groups can be easily distinguished based on nesting and habitat preferences. Species of the *aveli* species group inhabit lowlands and have nests inside dry stems of trees, bushes and herbs. While Greek species of the *tuberum* species group occur in the mountain areas and nest in soil, under moss, in rock crevices or debris.

*Temnothorax bulgaricus* species group: antennae 12-segmented, club usually distinctly darkened, metanotal groove absent, body colouration mostly yellow, sometimes head partly darkened, first gastral tergite with broad dark posterior band, always with yellow spot at base, propodeal spines reduced to triangular tubercle, petiole node rounded in profile, head in central part more or less smooth and shiny, on sides with longitudinal ridges, sometimes almost whole surface of head smooth and shiny or smooth area reduced to a narrow medial stripe, mesosoma laterally with strong sculpture of longitudinal ridges. In Greece two species associated with humid habitats such as stream valleys or bushes inside dark deciduous forests, *T. bulgaricus* (Forel) and *T. nadigi* (Kutter).

*Temnothorax clypeatus* species group: antennae 12-segmented, completely yellow, metanotal groove inconspicuous but well-marked, body colouration ochraceous to reddish brown, first gastral tergite with broad dark posterior band, always with pale spot at base, propodeal spines moderate to long, thorn-shaped, petiole with short peduncle, appears high and bulky in profile, head sculpture from complete, with microreticulate background and more or less developed ridges and costae, to mostly smooth and shiny in central part, only on gena and around eyes with longitudinal ridges, mesosoma laterally with distinct microreticulation and strong reticulate and longitudinal sculpture. In Greece one arboreal species associated with deciduous trees, especially large oaks in sunny habitats, *T. clypeatus* (Mayr).

*Temnothorax corticalis* species group: antennae 12-segmented, completely yellow, metanotal groove absent, body colouration ochraceous to light brown, head always more or less darkened, first gastral tergite with broad dark posterior band, always with pale spot at base, propodeal spines from very short, triangular to moderately long needle-shaped, petiole with very short peduncle, appears triangular in profile with more or less angulate node, head sculpture from complete, with more or less reticulate sculpture but shiny background, occasionally in central part of frons reticulation partly diffused, mesosoma dorsally and laterally with distinct microreticulation and often sides of pronotum with longitudinal ridges. In Greece one arboreal species associated with deciduous forests, *T. corticalis* (Schenck), but occurrence of *T. jailensis* (Arnoldi) is possible.

*Temnothorax exilis* species group: antennae 12-segmented, club usually distinctly darkened, metanotal groove absent, body colouration extremely variable, from almost completely yellow to black, often mesosoma paler coloured than head and gaster, first gastral tergite in pale forms with dark posterior band, propodeal spines moderate to long, thin, straight, petiole node angulate in profile, head in central part more or less smooth and shiny, on sides usually with longitudinal ridges, sometimes almost whole surface of head smooth and shiny or smooth area reduced to a narrow medial stripe, mesosoma laterally with strong sculpture of longitudinal ridges. Xerothermophilous species associated with rocky, open and arid habitats; in Greece only *T. exilis* (Emery) recorded, but based on a high variability of insular populations the real number of species of this group is difficult to estimate and requires further studies.

*Temnothorax flavicornis* species group: antennae 11-segmented, unicolourous yellow, metanotal groove present, body colouration almost completely yellow to dark yellow, without distinct contrast between colouration of head and mesosoma, first gastral tergite with dark posterior, propodeal spines from long to very long, claw-shaped, from straight to slightly curved, petiole with moderately long peduncle and node angulate, head always with microreticulate background and more or less developed reticulate or costulate sculpture, along middle of head runs stripe with diffused reticulation, more or less smooth and shiny, mesosoma with microreticulate sculpture, often with distinct ridges or costae. One species associated with various arboreal habitats, *T. flavicornis* (Emery).

*Temnothorax graecus* species group: antennae 12-segmented, club usually distinctly darkened, metanotal groove absent, body colouration mostly yellow, first gastral tergite with broad dark posterior band, always with yellow spot at base, propodeal spines very short to short, triangular to needle-shaped, petiole node rounded in profile or obtusely angulate, head in central part more or less smooth and shiny, on sides with longitudinal ridges, sometimes almost whole surface of head smooth and shiny or smooth area reduced to a broad medial stripe, mesosoma laterally with moderate sculpture of longitudinal ridges. Associated mostly with moderately humid to arid deciduous forests or mediterranean bushes, collected on rocks and stones; *T. aeolius* (Forel), *T. graecus* (Forel), *T. smyrnensis* (Forel), and at least two undescribed species.

*Temnothorax interruptus* species group: antennae 12-segmented, club usually distinctly darkened, frontal lobes conspicuously wider than frons, metanotal groove absent or indistinct, body yellow to light brown, gena darker, first gastral tergite with dark posterior band, often interrupted in the middle, propodeal spines very long, thin and curving downwards, petiole node subangulate to obtuse in profile, head with distinct microreticulation and longitudinal ridges, often partly with large reticulate sculpture, mesosoma mostly microreticulate, dorsally and laterally more or less rugose or costulate. In Greece two species associated with open habitats such as rocks and stones overgrown by bushes or limestones on mountain pastures, collected also in deciduous or mixed forests, and occasionally in coniferous forests; *T. morea* Csősz, Salata & Borowiec and *T. strymonensis* Csősz, Salata & Borowiec.

*Temnothorax kemali* species group: antennae 12-segmented, club usually more or less darkened, occasionally whole antennae yellow, metanotal groove absent, body yellow to orange, gena usually darker, first gastral tergite with dark posterior band, propodeal spines long and thin apically often curving downwards, petiole node subangulate to obtuse in profile, head at least in central part without microreticulation, smooth and shiny, only gena and area around eyes with longitudinal ridges, in some specimens sculpture of sides and central part of head more distinct but area between ridges or costae always smooth and shiny. Species associated with Mediterranean herbs and bushes or dry deciduous and coniferous forests, often nestling inside dry stems of herbs; *T. kemali* (Santschi) and at least one undescribed species.

*Temnothorax luteus* species group: antennae 12-segmented, club usually more or less darkened, occasionally whole antennae yellow, metanotal groove absent, body yellow to orange, gena sometimes darker, first gastral tergite with dark posterior band, propodeal spines straight, long and thin, petiole node subangulate in profile, head most often without microreticulation, smooth and shiny, only gena and area around eyes with longitudinal ridges. Xerothermophilous species associated with lowland habitats, Mediterranean herbs and bushes; at least one undescribed species.

*Temnothorax nylanderi* species group: diverse and speciose group, antennae 12-segmented, unicolourous yellow, metanotal groove usually distinct but in some species inconspicuous, body colouration variable, from almost completely yellow to dark brown but usually without distinct contrast between colouration of head and mesosoma, first gastral tergite usually with dark posterior band (except dark species with unicolourous gaster), propodeal spines from very short, triangular to very long, claw-shaped, from straight to distinctly curved, petiole with moderately long peduncle and node from angulate to obtuse in profile, head always with microreticulate background and more or less developed reticulate or costulate sculpture, sometimes along middle of head runs stripe with diffused reticulation, more or less smooth and shiny, mesosoma with microreticulate sculpture, often with distinct ridges or costae. Species associated with various shadowy habitats, nesting in rock, stones, and dry branches inside forests; *T. angulinodis* Csősz, Heinze & Mikó, *T. angustifrons* Csősz, Heinze & Mikó, *T. ariadnae* Csősz, Heinze & Mikó, *T. crasecundus* Seifert & Csősz, *T. crassispinus* (Karavaiev), *T. helenae* Csősz, Heinze & Mikó, *T. laconicus* Csősz, Seifert, Müller, Trindl, Schulz & Heinze, *T. lichtensteini* (Bondroit), *T. lucidus* Csősz, Heinze & Mikó, *T. nylanderi* (Foerster), *T. parvulus* (Schenck), *T. sordidulus* (Müller), *T. subtilis* Csősz, Heinze & Mikó, *T. tergestinus* (Finzi), and *T. triangularis* sp. nov.

Comment: Results presented by Prebus (2017) revealed that the *nylanderi* species group, as defined by Csősz et al. 2015, is paraphyletic and the position of *T. flavicornis* within it is unlikely. Therefore, we decided to exclude this species from this group and consider it as the single representative of the *flavicornis* species group.

*Temnothorax recedens* species group: antennae 12-segmented, unicolourous yellow to brown, metanotal groove very deep, body colouration variable, from almost completely yellow to dark brown, often mesosoma paler coloured than head and gaster, first gastral tergite in pale forms with dark posterior band, propodeal spines from short, triangular to long and thin, the shape of the needle, straight to slightly curved, petiole with long peduncle and node obtuse in profile, head mostly smooth and shiny, pronotum almost completely shiny, mesonotum and propodeum laterally with microreticulate sculpture, without distinct ridges or costae. Species associated with various arboreal habitats, nesting in rock crevices or under moss; *T. antigoni* (Forel), *T. recedens* (Nylander), *T. rogeri* Emery, and *T. solerii* (Menozzi).

Comment: With great probability this group is more speciose than it is apparent from current data, especially *T. recedens* shows high regional variability and wide ecological variance, which suggests that this taxon is a group of cryptic species

*Temnothorax rottenbergi* species group: very large species, antennae 12-segmented, club darkened or whole antennae dark, metanotal groove inconspicuous, body completely black or distinctly bicoloured with head and gaster brown to black and mesosoma partly to completely red, propodeal spines very long and strong, apex of spines often curving downwards, petiole with long peduncle and globular node, head with strong reticulate sculpture, mesosoma dorsally and laterally with strong, partly reticulate and partly costate sculpture. Xerothermophilous and alpine species associated with rock and stones on open, sunny habitats such as mountain pastures, grasslands or edges of forests. From Greece *T. rottenbergi* (Emery) and *T. semiruber* (André) were recorded but occurrence of first species needs confirmation.

*Temnothorax tuberum* species group: diverse group, antennae 12-segmented, club darkened, metanotal groove absent or indistinct, mesosoma colouration variable, from almost completely yellow to ochraceous, head always darker coloured than mesosoma, in extreme case almost black, first gastral tergite always with dark posterior band, propodeal spines from short, triangular to moderately long but never needle-shaped, from straight to slightly curved apically, petiole with moderately long peduncle and node subangulate in profile, head always with microreticulate background and more or less developed reticulate or costulate sculpture, without smooth and shiny areas, mesosoma with microreticulate sculpture, often with distinct ridges or costae – species associated with various habitats, from open and sunny to shadowy arboreal, usually nesting in rocks or stones, most species were noted also on rocks on mountain pastures – *T. melanocephalus* (Emery), *T. nigriceps* (Mayr), *T. tuberum* (Fabricius), *T. unifasciatus* (Latreille), and several undescribed taxa.

Comment: Results presented by Prebus (2017) suggest that the *tuberum* species group (sensu Cagniant and Espadaler 1997) and *unifasciatus* species group (sensu Bernard 1967) are paraphyletic. In both cases species groups were defined based on West-Mediterranean taxa. Only two Greek members of the *tuberum* species group were included in analysis presented by Prebus (2017): *T. nigriceps* and *T. unifasciatus* and they created a separate cluster. Confirmation, if the *tuberum* species group as defined by us here is a natural, monophyletic group requires further studies.

### A key to *Temnothorax* species groups known from Greece*

**Table:** 

1	Metanotal groove present, distinct to inconspicuous	**2**
–	Metanotal groove absent, mesosoma in lateral view evenly convex	7**
2	Antennae 11-segmented	***T. flavicornis* species group**
–	Antennae 12-segmented	**3**
3	Head and mesosoma almost entirely smooth and shiny; metanotal groove very deep; antennal scape very long and thin	***T. recedens* species group**
–	Head and mesosoma sculptured, sometimes only frons and mesosomal dorsum with reduced or absent sculpture; metanotal groove deep to indistinct; antennal scape short to moderate	**4**
4	More or less large species; body completely black or distinctly bicoloured with head and gaster brown to black and mesosoma partly to completely red; propodeal spines very long and sharp, apex of spines often curving downwards; petiole with long and more or less thin peduncle and globular node, head with sparse and thick reticulae and, at least on frons, smooth interspaces	***T. rottenbergi* species group**
–	Moderately sized species; body colouration variable, from almost completely yellow to dark brown but without distinct contrast between colouration of head and mesosoma; propodeal spines short to long, straight or only slightly curved; petiole with peduncle short to moderately long, and node from angulate to obtuse in profile; head with sculpture variable, but always fine and dense, interspaces usually with additional microsculpture	**5**
5	Promesonotal suture distinct; body colouration ochraceous to reddish brown; anterior margin of clypeus with distinct notch	***T. clypeatus* species group**
–	Promesonotal suture absent or indistinct; body yellow to dark brown and never ochraceous or reddish; anterior margin of clypeus without distinct notch	**6**
6	Antennal club darkened; metanotal groove feebly marked; body colouration more or less uniform, mostly brown to almost black; first gastral tergite with yellowish brown basal spot; propodeal spines short to long but thin, straight or only slightly curved	***T. angustulus* species group**
–	Antennal club unicolourous, yellow; metanotal groove usually distinct but in some species inconspicuous; body colouration variable, from almost completely yellow to dark brown but never uniformly coloured (at least legs and antennae brighter); first gastral tergite with dark posterior band or uniformly coloured; propodeal spines from very short, triangular to very long, claw-shaped, always with wide base	***T. nylanderi* species group**
7	Frontal lobes conspicuously wider than frons; first gastral tergite with dark posterior band, often interrupted in the middle; propodeal spines very long, thin and curving downwards	***T. interruptus* species group**
–	Frontal lobes never conspicuously wider than frons; dark posterior band on the first gastral tergite never interrupted in the middle or absent; propodeal spines of a different shape	**8**
8	Propodeal spines very long and thin and never with wide base	**9**
–	Propodeal spines very short to moderately long, always with wide base	**11**
9	Body orange to dark orange with darker head and first gastral tergite with bright spot on its basal part; head and mesosoma surface moderately sculptured, head completely microsculptured and with more or less developed longitudinal ridges	***T. affinis* species group**
–	Body yellow to orange, gena sometimes darker, first gastral tergite with dark posterior band; head, at least in central part, without microreticulation, smooth and shiny	**10**
10	Propodeal spines long and thin, apically often curving downwards; head microsculptured, only its central part with microreticulation reduced or absent	***T. kemali* species group**
–	Propodeal spines straight, long and thin; head most often without microreticulation, smooth and shiny	***T. luteus* species group**
11	Body colouration uniform, brown, dark brown to almost black; propodeal spines very short with broad base, not longer than basal width; petiole node rounded in profile; head and mesosoma surface very strongly sculptured; alpine species	***T. anodontoides* species group**
–	Body bicoloured or entirely yellow; if body uniformly dark brown to black then propodeal spines small to moderately long, and frons and dorsal surface of mesosoma with reduced sculpture or smooth	**12**
12	Propodeal spines indistinct, strongly reduced to triangular tubercles; petiolar node obtuse in profile	***T. bulgaricus* species group**
–	Propodeal spines short to moderately long, always distinct; petiolar node from obtuse to angulate in profile	**13**
13	Head sculpture variable, more or less sparse and fine; at least centre of frons and central part of promesonotal dorsum smooth; propodeal spines moderately long to long; body colouration variable, from almost completely yellow to completely black; also within populations of the same species body colouration variable	***T. exilis* species group**
–	Head with dense and fine sculpture, frons sometimes with sculpture reduced but never smooth; sculpture on mesosoma never reduced or smooth. If head and dorsum of promesonotum with reduced sculpture, then propodeal spines very small; body colouration from mostly yellow to light brown or bicoloured but constant within a single species	**14**
14	Body distinctly bicoloured, yellow to light brown with head usually darker than mesosoma and gaster with broad dark band	**15**
–	Body almost entirely yellow to dark yellow; only head sometimes with slightly darker frons or malar area and gaster usually with narrow dark band	**16**
15	Antennal club never darkened; propodeal spines from very short, triangular to moderately long needle-shaped, petiole with very short pedicel; head with moderately dense reticulate sculpture but smooth background, occasionally in central part of frons reticulation partly diffused	***T. corticalis* species group**
–	Antennal club darkened; propodeal spines from short, triangular to moderately long but never needle-shaped; petiole with moderately long pedicel; head always with microreticulate background and moderately developed reticulate or costulate sculpture, without smooth and shiny areas	***T. tuberum* species group**
16	Propodeal spines short to moderately long with broad base; head and mesosoma surface finely sculptured, head almost uniformly, regularly microsculptured, mostly without longitudinal ridges, sometimes only central part of head with narrow area of diffused microreticulation, but head never appears smooth and shiny; petiolar node angulate in profile	***T. aveli* species group**
–	Propodeal spines very short to short, triangular to needle-shaped; head in central part more or less smooth and shiny, on sides with longitudinal ridges, sometimes almost whole surface of head smooth and shiny or smooth area reduced to a broad medial stripe; petiolar node rounded to subangular in profile	***T. graecus* species group**

### Review of Greek species of the *Temnothorax aveli* species group

#### Temnothorax brackoi sp. nov.

Taxon classificationAnimaliaHymenopteraFormicidae

EFFC3F26-765C-575C-A7D3-F99B7483F11D

http://zoobank.org/BAAA6DC9-5DB0-4A21-8520-679D413E3BC6

Figs 1 , 2 , 9 , 13

##### Type material.

**Holotype**: worker (pin), (CASENT0846669): GREECE, Pel. Messinia | 1.8 km E of Saidona, 985 m | 36.88491N, 22.30419E | 20 VI 2016, L. Borowiec || Collection L. Borowiec | Formicidae | LBC-GR02195 (MNHW).

**Paratypes**: • 11w. (pin), 9w. (EtOH) (CASENT0846607-CASENT0846617): the same nest sample as holotype (DBET); • 11w. (pin), 5w. (EtOH)(CASENT0846618-CASENT0846628): GREECE, Pel. Messinia | 0.8 km SE of Exochori, 535 m, | 36.89582N, 22.27464E | 20 VI 2016, L. Borowiec || Collection L. Borowiec | Formicidae | LBC-GR02184 (DBET, MHNG, CASC, BMNH); • 8w. (pin), 1w. (EtOH) (CASENT0846629-CASENT0846636): GREECE, Pel. Messinia | n. Arachova, 860 m | 37.03922N, 22.21876E | 13 VI 2016, L. Borowiec || Collection L. Borowiec | Formicidae | LBC-GR02029 (DBET); • 2w. (pin) (CASENT0846637-CASENT0846638): GREECE, Pel. Messinia | 1.3 km S of Artemisia, 870 m | 37.08738N, 22.23378E | 17 VI 2016, L. Borowiec || Collection L. Borowiec | Formicidae | LBC-GR02121 (DBET).

##### Other material.

**GREECE. Epirus, Preveza**: • 4w. (pin) (CASENT0846670-CASENT0846673), 21w. (EtOH): 700 m E of Kanali, oak forest, 39.0638N/20.70621E, 50 m, 2016-08-27, leg. L. Borowiec; • 8w. (pin) (CASENT0846674-CASENT0846681), 14w. (EtOH): n. Kanali, oak forest, 39.05458N/20.70207E, 15 m, 2016-08-28, leg. L. Borowiec; • 27w (EtOH): n. Kanali, pine forest, 39.05388N/20.70118E, 20 m, 2016-08-28, leg. L. Borowiec. **Ionian Islands, Cephalonia**: • 6w. (pin) (CASENT0846682-CASENT0846687): Avithos Lake, area near a small lake in a moist, shaded valley of a small creek, 38.17293N/20.71233E, 278 m, 2014-06-25, leg. L. Borowiec; • 68w. (EtOH): Avithos Lake, shrubs around small lake, 38.17203N/ 20.71107E, 288 m, 2019-06-10, leg. L. Borowiec; • 29w. (EtOH): Katapodata; roadsides with shrubs, 38.23337N/20.64594E, 100 m, 2019-06-10, leg. L. Borowiec; • 4w. (EtOH): 1.5 km NE of Koulourata, mixed forest on shrubs, 38.20667N/ 20.67715E, 273 m, 2019-06-10, leg. L. Borowiec; • 11w. (EtOH): ancient Same; roadsides with shrubs, 38.2522N/20.66423E, 220 m, 2019-06-10, leg. L. Borowiec; • 13w. (EtOH): rd. Skala-Poros; Mediterranean shrubs, 38.12872N/20.79576E, 5 m, 2019-06-12, leg. L. Borowiec. **Ionian Islands, Corfu**: • 6w. (EtOH): Ag. Ilias, deciduous forest, 39.79367N/19.87508E, 191 m, 2013-06-10, leg. L. Borowiec; • 5w. (pin) (CASENT0846688-CASENT0846692), 5w. (EtOH): Akr. Kefali n. Agios Stefanos, frygana on sea coast, 39.75154N/19.63272E, 13 m, 2013-06-05, leg. L. Borowiec; • 4w. (EtOH): E of Nymfes, old olive tree plantation, 39.75047N/19.8057E, 179 m, 2013-06-09, leg. L. Borowiec; • 8w. (pin) (CASENT0846693-CASENT0846700), 1w. (EtOH): Klimatia, deciduous forest, 39.74123N/19.78953E, 311 m, 2013-06-06, leg. L. Borowiec; • 1w. (pin) (CASENT0846701): N of Ag. Stefanos, old olive forest, 39.76338N/19.65213E, 88 m, 2013-06-05, leg. L. Borowiec; • 4w. (pin) (CASENT0846702-CASENT0846705): n. Doukades, old olive tree plantation, 39.70075N/19.75055E, 174 m, 2013-06-08, leg. L. Borowiec; • 3w. (pin) (CASENT0846706-CASENT0846708), 1w. (EtOH): n. Vistonas, old olive tree plantation, 39.68549N/19.71453E, 422 m, 2013-06-08, leg. L. Borowiec; • 20w. (pin) (CASENT0846709-CASENT0846728), 15w. (EtOH): Nymfes, deciduous forest, 39.75478N/19.79535E, 162 m, 2013-06-06, leg. L. Borowiec; • 20w. (EtOH): Old Perithia, old mountain village, 39.76159N/19.87412E, 467 m, 2013-06-10, leg. L. Borowiec; • 3w. (pin) (CASENT0846729-CASENT0846731): Strinilas, deciduous forest, 39.73963N/19.84265E, 632 m, 2013-06-07, leg. L. Borowiec. **Ionian Islands, Lefkada**: • 3w. (pin) (CASENT0846732-CASENT0846734), 2w. (EtOH): Asprogerakata, stream valley with deciduous forest, 38.78278N /20.65262E, 430 m, 2016-09-02, leg. L. Borowiec; • 2w. (pin) (CASENT0846735-CASENT0846736), 8w. (EtOH): Egklouvi, stream valley with deciduous forest, 38.73212N/20.64821E, 690 m, 2016-09-02, leg. L. Borowiec. **Ionian Islands, Zakynthos**: • 4w. (EtOH): 1 km E of Elies, shrubs along roadsides, 37.90173N/20.68787E, 300 m, 2018-05-06, leg. L. Borowiec; • 18w. (EtOH): 1 km N of Exo Chora, mixed forest, 37.81063N/20.68459E, 430 m, 2018-05-08, leg. L. Borowiec; • 1w. (pin) (CASENT0846737): 1.2 km N of Ano Vasilikos, roadsides along olive plantation and pasture with oak shrubs, 37.72456N/20.97786E, 30 m, 2018-05-05, leg. L. Borowiec; • 128w. (EtOH): 1.2 km N of Vasilikos, roadsides along olive plantation and pasture with oak shrubs, 37.72456N/20.97786E, 30 m, 2018-05-05, leg. L. Borowiec; • 2w. (EtOH): 1.2 km NE of Anafonitria shrubs around burned forests, 37.85489N/20.64124E, 475 m, 2018-05-10, leg. L. Borowiec; • 1w. (pin) (CASENT0846738), 43w. (EtOH): 1.2 km SW of Ano Vasilikos loc. 1, roadsides along olive plantation, 37.72374N/20.95964E, 72 m, 2018-05-05, leg. L. Borowiec; • 1w. (pin) (CASENT0846739), 25w. (EtOH): 1.2 km SW of Ano Vasilikos loc. 2, roadsides along olive plantation, 37.72325N/20.96123E, 75 m, 2018-05-05, leg. L. Borowiec; • 1w. (pin): 1.2 km SW of Skinaria, limestone hills after burned forests, 37.87694N/20.69272E, 375 m, 2018-05-06, leg. L. Borowiec; • 1w. (pin) (CASENT0846740), 13w. (EtOH): 1.4 km E of Ano Volimes, mixed forest, 37.87594N/20.68525E, 430 m, 2018-05-06, leg. L. Borowiec; • 1w. (EtOH): 1.5 km N of Exo Chora: shrubs around meadow, 37.81446N/ 20.68826E, 460 m, 2018-05-08, leg. L. Borowiec; • 38w. (EtOH): 1.7 km NE of Ano Volimes, shrubs around pastures, 37.88627N/20.68456E, 445 m, 2018-05-06, leg. L. Borowiec; • 3w. (EtOH): 1.8 km SW of Volimes, shrubs along roadsides, 37.86472N/20.64234E, 350 m, 2018-05-10, leg. L. Borowiec; • 1w. (EtOH): 1.9 km W of Maries, roadsides in burned forests, 37.818N/20.65556E, 290 m, 2018-05-09, leg. L. Borowiec; • 1w. (pin), 30w. (EtOH): 2.5 km NE of Maries, mixed forest, 37.8299N/20.70151E, 475 m, 2018-05-08, leg. L. Borowiec; • 51w. (EtOH): 3.9 km NE of Maries, shrubs around olive plantation, 37.84202N/20.70938E, 370 m, 2018-05-08, leg. L. Borowiec; • 1w. (pin) (CASENT0846741), 16w. (EtOH): 470 m NE of Orthonies, shrubs along roadsides, 37.85435N/20.69843E, 405 m, 2018-05-10, leg. L. Borowiec; • 1w. (EtOH): 500 m S of Apelati, pine forest, 37.66873N/20.81407E, 160 m, 2018-05-07, leg. L. Borowiec; • 1w. (EtOH): 700 m SW of Koroni, maquis, 37.86582N/20.71753E, 290 m, 2018-05-10, leg. L. Borowiec; • 3w. (EtOH): 750 m S of Volimes, shrubs in cypress forest, 37.86762N/20.66191E, 360 m, 2018-05-10, leg. L. Borowiec; • 1w. (pin) (CASENT0846742), 81w. (EtOH): 800 m SE of Xirokastello, roadsides along olive plantation, 37.73491N/20.95139E, 75 m, 2018-05-05, leg. L. Borowiec; • 10w. (EtOH): 880 m S of Orthonies, shrubs in cypress forest, 37.84462N/20.69843E, 390 m, 2018-05-10, leg. L. Borowiec; • 66w. (EtOH): Ag. Georgiou monastery, shrubs along roadsides, 37.85971N/20.63646E, 330 m, 2018-05-10, leg. L. Borowiec; • 8w. (EtOH): Ag. Joannis, roadsides with shrubs, 37.72924N/20.94553E, 165 m, 2018-05-05, leg. L. Borowiec; • 6w. (EtOH): Argassi, urban area, 37.76182N/20.92704E, 10 m, 2018-05-04, leg. L. Borowiec; • 1w. (EtOH): Livia Mts. loc. 1, shrubs along roadsides, 37.83018N/20.71619E, 600 m, 2018-05-08, leg. L. Borowiec; • 15w. (EtOH): Vrachionas Mts., mountain pastures with shrubs, 37.81798N/ 20.70621E, 670 m, 2018-05-08, leg. L. Borowiec; • 1w. (EtOH): W of Kampi, pine forest, 37.78161N/20.68078E, 165 m, 2018-05-09, leg. L. Borowiec. **Macedonia, Pieria**: • 4w. (EtOH): Platamonas Castle hill, on stones, soil and herbs, 40.00868N/ 22.59654E, 12 m, 2019-05-11, leg. L. Borowiec; • 1w. (pin) (CASENT0846743): 2 km W of Panteleimonas, roadsides with shrubs, 39.98563N/ 22.59513E, 305 m, 2019-05-15, leg. L. Borowiec; • 20w. (EtOH): road to P. Poroi loc. 1, roadsides with shrubs, 39.97963N/ 22.61563E, 110 m, 2019-05-17, leg. L. Borowiec; • 7w. (EtOH): road to P. Poroi loc. 2, roadsides with shrubs, 39.97627N/ 22.61146E, 185 m, 2019-05-17, leg. L. Borowiec; •81w. (EtOH): road to P. Poroi loc. 3, roadsides with shrubs, 39. 96863N/22.60494E, 260 m, 2019-05-17, leg. L. Borowiec. **Peloponnese, Korinthia**: • 16w. (pin) (CASENT0846744-CASENT0846759): n. Evrostina, deciduous forest, 38.07386N/22.39388E, 662 m, 2013-09-01, leg. L. Borowiec. **Peloponnese, Laconia**: • 13w. (pin) (CASENT0846760-CASENT0846772), 2w. (EtOH): Taygetos Mts., 1.5 km SW of Anavryti, coniferous forest, 37.0191N/22.36003E, 990 m, 2016-06-21, leg. L. Borowiec. **Peloponnese, Messinia**: • 1w. (pin) (CASENT0846773): Taygetos Mts., 0.7 km S of Dyrrachio, coniferous forest, 37.1811N/22.2074E, 800 m, 2016-06-22, leg. L. Borowiec; • 5w. (pin) (CASENT0846774-CASENT0846778): Taygetos Mts., 2 km W of Arachova, stream valley with Platanus forest, 37.0357N/22.1978E, 680 m, 2016-06-13, leg. L. Borowiec; • 1w. (pin) (CASENT0846779): Taygetos Mts., Chora Getson, stream valley with deciduous forest, 36.94779N/22.25466E, 615 m, 2016-06-14, leg. L. Borowiec; • 4w. (pin) (CASENT0846780-CASENT0846783): Taygetos Mts., Karveli, rest area with stream, 37.07591N/22.20633E, 600 m, 2016-06-17, leg. L. Borowiec; • 2w. (pin) (CASENT0846784-CASENT0846785): Tetrazi Mts., 0.5 km E of Vastas, stream valley with deciduous forest, 37.36441N/21.99304E, 895 m, 2016-06-19, leg. L. Borowiec; • 1w. (pin) (CASENT0846786), 2w. (EtOH): Tetrazi Mts., Isaris, pine forest, 37.36644N/22.01516E, 790 m, 2016-06-19, leg. L. Borowiec; • 1w. (pin) (CASENT0846787): Tetrazi Mts., Karnasi, stream valley with Platanus forest, 37.31904N/21.99158E, 460 m, 2016-06-19, leg. L. Borowiec. **Sterea Ellas, Aetolia-Acarnania**: • 1w. (EtOH): Psila Alonia, stream valley with Platanus forest, 38.96255N/21.19527E, 62 m, 2016-09-04, leg. L. Borowiec. **Sterea Ellas, Euboea**: • 2w. (EtOH): 1.2 km NW of Gerontas, mediterranean shrubs along roadsides, 38.45885N/23.808E, 405 m, 2018-06-10, leg. L. Borowiec; • 2w. (EtOH): 2.3 km S of Stropones, mixed forest, 38.9933N/23.87807E, 860 m, 2018-06-10, leg. L. Borowiec; • 1w. (EtOH): 300 m NW of Agios, pine forest with mediterranean shrubs, 38.65856N/23.55525E, 600 m, 2018-06-11, leg. L. Borowiec; • 7w. (EtOH): 570 m NW of Drosia, stream valley with mixed forest, 38.61705N/23.59089E, 140 m, 2018-06-11, leg. L. Borowiec. **Thessaly, Larissa**: • 1w. (pin) (CASENT0846788): Mt. Olympus, 5.3 km E of Olympiada, alpine pastures with shrubs, 40.00989N/22.31096E, 800 m, 2017-05-09, leg. L. Borowiec; • 7w. (EtOH): Mt. Ossa, 2.4 km SE of Karitsa, stream valley in deciduous forest, 39.82632N/22.77557E, 425 m, 2017-05-04, leg. L. Borowiec; • 12w. (EtOH): Mt. Ossa, 600 m SE of Karitsa, stream valley in deciduous forest, 39.84009N/22.76983E, 300 m, 2017-05-04, leg. L. Borowiec; • 3w. (pin) (CASENT0846789-CASENT0846791), 44w. (EtOH): Mt. Ossa, Kokkino Nero, stream valley in urban area, 39.83389N/22.79379E, 3 m, 2017-05-10, leg. L. Borowiec. **CROATIA, Podgorje**: • 4w. (pin) (CASENT0846792-CASENT0846795), vicinity Sibenj, 9 km S Senj, 0–50 m, 3.6.1997, A. Schulz & K. Vock leg.

##### Locus typicus.

Peloponnese, Taygetos Mts.

##### Differential diagnosis.

*Temnothorax brackoi* is most similar to *T. aveli* (Bondroit, 1918), a species widely distributed in the western part of the Mediterranean basin, east to Slovenia. It is similar to *T. aveli* especially in body shape, petiolar structure and unicolour yellow antennae. *Temnothorax aveli* differs in microreticulation of head not as regular as in *T. brackoi*, with slightly shiny surface between sculpture and often with presence of median stripe of diffused microsculpture on frons, while in *T. brackoi* head sculpture is always homogenous, without areas of diffused microreticulation and dull surface between sculpture. From Greek members of *Temnothorax*, *T. brackoi* is most similar to sympatric *T. graecus*, but differs from it in well-developed sculpture on the whole head surface (in *T. graecus* central part of frons is more or less shiny, without microreticulation) and larger, distinctly triangular propodeal spines (in *T. graecus* spines are short, needle-shaped); some specimens of *T. brackoi* can also be confused with species belonging to the *T. tuberum* group, but *T. brackoi* differs from them in low and arched petiolar node and lack of longitudinal striation on frons.

##### Description.

Worker (n = 10): HL: 0.637 ± 0.04 (0.584–0.683); HW: 0.547 ± 0.03 (0.509–0.696); SL: 0.437 ± 0.02 (0.410–0.560); EL: 0.148 ± 0.01 (0.124–0.174); EW: 0.105 ± 0.01 (0.087–0.124); WL: 0.735 ± 0.05 (0.671–0.820); PSL: 0.155 ± 0.015 (0.130–0.174); SDL: 0.111 ± 0.01 (0.096–0.124); PEL: 0.271 ± 0.03 (0.224–0.311); PPL: 0.168 ± 0.01 (0.149–0.186); PEH: 0.208 ± 0.015 (0.186–0.236); PPH: 0.207 ± 0.015 (0.180–0.224); PNW: 0.368 ± 0.02 (0.329–0.401); PLW: 0.170 ± 0.01 (0.149–0.199); PPW: 0.223 ± 0.02 (0.196–0.242); CI: 85.9 ± 1.2 (83.1–87.5); SI1: 68.6 ± 1.6 (66.7–71.3); SI2: 79.9 ± 1.6 (77.1–81.9); MI: 50.0 ± 1.0 (48.4–51.3); EI1: 71.0 ± 5.0 (64.6–78.3); EI2: 16.5 ± 1.4 (14.8–18.75); PI: 130.0 ± 7.9 (120.0–145.5); PPI: 81.4 ± 6.1 (128.2–150.0); PSI: 135.8 ± 12.4 (120.0–155.6).

##### Colour.

**Figures 1, 2.**
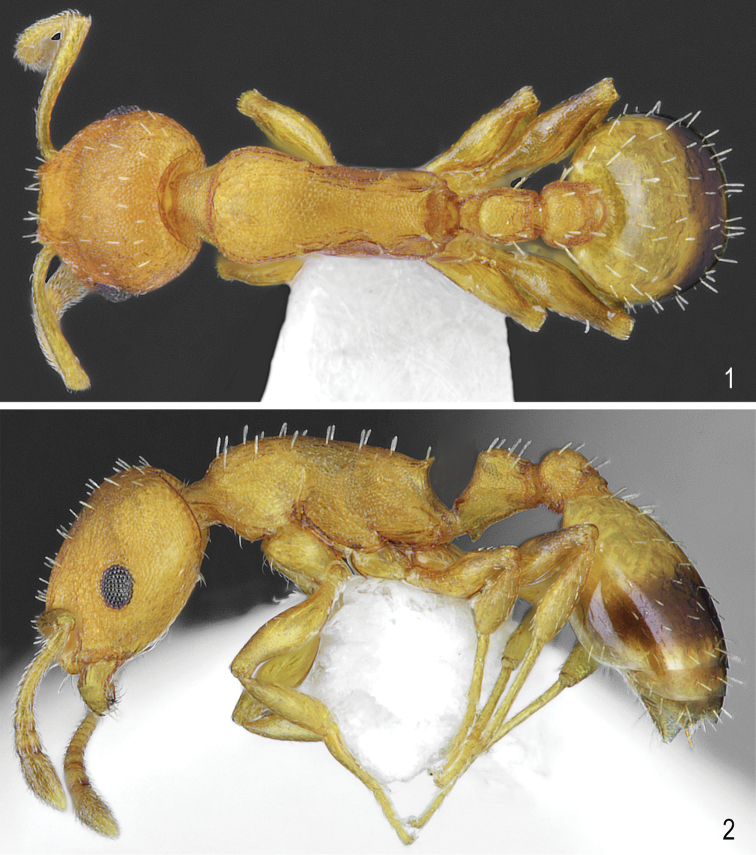
Worker of *Temnothorax brackoi* sp. nov. **1** Dorsal **2** Lateral.

Head, antennae, mesosoma, petiole, postpetiole and legs uniformly yellow to dark yellow, in few specimens, frons and femora partly darkened. Gaster yellow, only the first gaster tergite with wide, dark band on its posterior part (Figs 1, 2). **Head.** Rectangular, 1.16 times as long as wide, lateral surfaces below and above eyes gently convex, posterior edges convex, occipital margin of head straight or slightly concave (Figs 9, 13). Anterior margin of clypeus slightly convex, medial notch absent. Eyes moderate, oval, 1.4 times as long as wide. Antennal scape short, in lateral view slightly curved, 0.69 times as long as length of the head, in apex gradually widened, its base with small, triangular tooth, funiculus long, club 3-segmented (Fig. 9). Surface of scape with very fine microreticulation, shiny, covered with thin, moderate dense, decumbent to suberect setae. Mandibles rounded with thick sparse, longitudinal striae, shiny. Clypeus shiny with thick, sparse, longitudinal striae, area between striae smooth and shiny. Frontal carinae short, not extending beyond frontal lobes. Antennal fossa deep, with sparse, thin, roundly curved striae and dense reticulation. Frontal lobes narrow, smooth with slight, dense longitudinal striation (Fig. 13). Frons, vertex and temples with dense, thick, reticulation, sometimes central surface of frons with additional a few thin, longitudinal, interrupted wrinkles, surface between striation smooth; malar area with irregular, thick, reticulation, space between reticulation smooth or with very sparse microreticulation, shiny; genae with sparser, thank of frons, and thick reticulation, shiny. Frons and vertex with erect, pale, short and thick setae (Fig. 13). **Mesosoma.** Elongate, approximately twice as long as wide, slightly arched in profile. Metanotal groove absent. Pronotum convex on sides. Propodeal spines short to moderate, triangular, directed upward, base wide, tips sharp (Fig. 2). Whole surface with dense, reticulation, its dorsal surface and lateral surfaces of pronotum with thick and sparse longitudinal striation or reticulation. Area between thick sculpture shiny, smooth or sometimes with sparse, fait microreticulation. Entire mesosoma bearing erect, pale, short and thick setae (Fig. 2). **Petiole.** In lateral view, with short peduncle, node low, with anterior face flat, and posterior face convex and dorsum convex. Peduncle and petiolar node shiny, with thick, dense reticulation, area between rugae smooth, dorsum with sparser reticulation. Dorsal surface bearing sparse, short, erect setae (Fig. 2). **Postpetiole.** In lateral view, regularly convex, apical half with gently convex sides (Figs 1, 2), on the whole surface shiny, with thick, dense reticulation, dorsum with sparser reticulation; area between rugae smooth. Dorsal surface bearing sparse, short, semierect to erect setae (Fig. 2). **Gaster.** Gaster smooth and shiny, bearing erect, thin, pale setae (Figs 1, 2).

##### Etymology.

Named after Gregor Bračko, a Slovenian myrmecologist, for his significant contributions to the studies on ants of the Balkan Peninsula.

##### General distribution.

Greece: Epirus, Ionian Islands, Peloponnese, western Sterea Ellas, Thessaly; Croatia: Dalmatia.

##### Biology.

Specimens of *T. brackoi* were collected from various localities: sunny and shadowy located on lowlands and highlands (9 - 990 m a.s.l). The species was noted in various habitats, most often on shrubs and trees growing along roadsides and olive plantations, maquis, phrygana, often inside forests (mostly deciduous, occasionally coniferous). We noted also its presence on the leaves of climbing plants growing on walls or trees. Nests were not found, probably like other species of this group, are located inside dry stems of herbs.

The following ant species were recorded in the same areas as *T. brackoi*:

**Epirus, Preveza, 700 m E of Kanali**: *Aphaenogaster balcanica* (Emery), *A. subterranea* (Latreille), *Camponotus dalmaticus* (Nylander), *C. fallax* (Nylander), *C. lateralis* (Olivier), *C. piceus* (Leach), *C. vagus* (Scopoli), *Cataglyphis nodus* (Brullé), *Colobopsis truncata* (Spinola), *Crematogaster schmidti* (Mayr), *Dolichoderus quadripunctatus* (Linnaeus), *Lasius lasioides* (Emery), *Messor wasmanni* Krausse, *Myrmecina graminicola* (Latreille), *Pheidole pallidula* (Nylander), *Plagiolepis pygmaea* (Latreille), *Temnothorax bulgaricus* (Forel), *T. rogeri* Emery; **near Kanali, oak forest**: *Aphaenogaster balcanica* (Emery), *A. subterranea* (Latreille), *Camponotus dalmaticus* (Nylander), *C. fallax* (Nylander), *C. lateralis* (Olivier), *Cataglyphis nodus* (Brullé), *Colobopsis truncata* (Spinola), *Crematogaster schmidti* (Mayr), *Lasius lasioides* (Emery), *Pheidole pallidula* (Nylander); **near Kanali, pine forest**: *Temnothorax bulgaricus* (Forel).

**Ionian Islands, Cephalonia, Avithos Lake**: *Aphaenogaster muelleriana* Wolf, *Camponotus dalmaticus* (Nylander), *C. gestroi* Emery, *C. ionius* Emery, *C. lateralis* (Olivier), *Crematogaster schmidti* (Mayr), *C. sordidula* (Nylander), *Lepisiota frauenfeldi* (Mayr), *Liometopum microcephalum* (Panzer), *Messor wasmanni* Krausse, *Monomorium monomorium* Bolton, *Myrmecina graminicola* (Latreille), *Pheidole pallidula* (Nylander), *Plagiolepis pygmaea* (Latreille), *Tapinoma festae* Emery, *Temnothorax bulgaricus* (Forel), *T. exilis* (Emery), *T. laconicus* Csősz et al., *Tetramorium kephalosi* Salata & Borowiec; **Katapodata**: *Camponotus dalmaticus* (Nylander), *Crematogaster schmidti* (Mayr), *Lepisiota frauenfeldi* (Mayr), *Plagiolepis pygmaea* (Latreille); **1.5 km NE of Koulourata**: *Aphaenogaster balcanica* (Emery), *Camponotus dalmaticus* (Nylander), *C. kiesenwetteri* (Roger), *C. lateralis* (Olivier), *Crematogaster schmidti* (Mayr), *Lepisiota frauenfeldi* (Mayr), *Pheidole balcanica* Seifert, *Plagiolepis pygmaea* (Latreille), *Temnothorax bulgaricus* (Forel), *T. leviceps* (Emery). *T. messiniaensis* sp. nov.; **ancient Same**: *Camponotus kiesenwetteri* (Roger), *C. lateralis* (Olivier), *Colobopsis truncata* (Spinola), *Crematogaster ionia* Forel, *C. sordidula* (Nylander), *Pheidole balcanica* Seifert, *Temnothorax bulgaricus* (Forel), *T. messiniaensis* sp. nov.; **road Skala-Poros**: *Camponotus aethiops* (Latreille), *C. gestroi* Emery; *C. dalmaticus* (Nylander), *C. kiesenwetteri* (Roger), *C. lateralis* (Olivier), *Colobopsis truncata* (Spinola), *Crematogaster schmidti* (Mayr), *C. sordidula* (Nylander), *Temnothorax messiniaensis* sp. nov.

**Ionian Islands, Corfu, Ag. Ilias**: *Aphaenogaster subterranea* (Latreille), *Camponotus dalmaticus* (Nylander), *C. lateralis* (Olivier), *Temnothorax laconicus* Csősz et al.; **Akr. Kefali near Agios Stefanos**: *Aphaenogaster balcanica* (Emery), *Bothriomyrmex communista* Santschi, *Camponotus aethiops* (Latreille), *C. dalmaticus* (Nylander), *C. lateralis* (Olivier), *C. piceus* (Leach), *Crematogaster schmidti* (Mayr), *C. sordidula* (Nylander), *Lasius illyricus* Zimmermann, *Lepisiota melas* (Emery), *L. nigra* (Dalla Torre), *Messor wasmanni* Krausse, *Pheidole pallidula* (Nylander), *Plagiolepis pygmaea* (Latreille), *Prenolepis nitens* (Mayr), *Temnothorax laconicus* Csősz et al.; **E of Nymfes**: *Aphaenogaster muelleriana* Wolf, *A. subterranea* (Latreille), *Camponotus aethiops* (Latreille), *C. dalmaticus* (Nylander), *C. lateralis* (Olivier), *Crematogaster sordidula* (Nylander), *Dolichoderus quadripunctatus* (Linnaeus), *Formica gagates* Latreille, *Lasius bombycina* Seifert & Galkowski, *L. illyricus* Zimmermann, *Lepisiota frauenfeldi* (Mayr), *Temnothorax laconicus* Csősz et al.; **Klimatia**: *Aphaenogaster balcanica* (Emery), *A. muelleriana* Wolf, *A. subterranea* (Latreille), *Camponotus aethiops* (Latreille), *C. dalmaticus* (Nylander), *C. gestroi* Emery, *C. lateralis* (Olivier), *C. heidrunvogtae* Seifert, *C. piceus* (Leach), *Cataglyphis nodus* (Brullé), *Colobopsis truncata* (Spinola), *Crematogaster schmidti* (Mayr), *C. sordidula* (Nylander), *Dolichoderus quadripunctatus* (Linnaeus), *Formica gagates* Latreille, *Lasius bombycina* Seifert & Galkowski, *L. illyricus* Zimmermann, *L. lasioides* (Emery), *Lepisiota frauenfeldi* (Mayr), *L. nigra* (Dalla Torre), *Messor ibericus* Santschi, *M. wasmanni* Krausse, *Myrmecina graminicola* (Latreille), *Pheidole pallidula* (Nylander), *Plagiolepis pygmaea* (Latreille), *P. xene* Stärcke, *Prenolepis nitens* (Mayr), Temnothorax cf. exilis, *T. laconicus* Csősz et al., *T. rogeri* Emery, *Tetramorium kephalosi* Salata & Borowiec.; **N of Ag. Stefanos**: *Aphaenogaster balcanica* (Emery), *Camponotus aethiops* (Latreille), *C. fallax* (Nylander), *C. lateralis* (Olivier), *C. piceus* (Leach), *Crematogaster schmidti* (Mayr), *Lasius bombycina* Seifert & Galkowski, *L. illyricus* Zimmermann, *Lepisiota frauenfeldi* (Mayr), *Messor ibericus* Santschi, *Pheidole pallidula* (Nylander), *Plagiolepis pygmaea* (Latreille), Solenopsis cf. fugax, *Temnothorax clypeatus* (Mayr); **near Doukades**: *Camponotus lateralis* (Olivier), *C. piceus* (Leach), *Lasius lasioides* (Emery); **near Vistonas**: *Camponotus aethiops* (Latreille), *C. dalmaticus* (Nylander), *C. piceus* (Leach), *Crematogaster sordidula* (Nylander), *Lepisiota melas* (Emery), *Messor ibericus* Santschi, *M. wasmanni* Krausse, *Plagiolepis pygmaea* (Latreille), Temnothorax cf. exilis, *T. rogeri* Emery, *Tetramorium kephalosi* Salata & Borowiec; **Nymfes**: Aphaenogaster cf. subterranea, *Temnothorax laconicus* Csősz et al., T. cf. tuberum, Tetramorium cf. caespitum; **Corfu, Old Perithia**: *Aphaenogaster balcanica* (Emery), *A. epirotes* (Emery), *A. muelleriana* Wolf, *Bothriomyrmex communista* Santschi, *Camponotus aethiops* (Latreille), *C. dalmaticus* (Nylander), *C. gestroi* Emery, *C. lateralis* (Olivier), *C. piceus* (Leach), *Crematogaster schmidti* (Mayr), *Lasius illyricus* Zimmermann, *L. lasioides* (Emery), *Lepisiota frauenfeldi* (Mayr), *L. melas* (Emery), *Messor ibericus* Santschi, *M. wasmanni* Krausse, *Plagiolepis pygmaea* (Latreille), *Prenolepis nitens* (Mayr), *Tapinoma erraticum* (Latreille), *Temnothorax affinis* (Mayr), T. cf. exilis, *T. laconicus* Csősz et al., T. cf. nigriceps, *T. rogeri* Emery, Tetramorium cf. caespitum, *T. kephalosi* Salata & Borowiec; **Strinilas**: *Aphaenogaster balcanica* (Emery), *A. epirotes* (Emery), *Bothriomyrmex communista* Santschi, *Camponotus dalmaticus* (Nylander), *C. lateralis* (Olivier), *Formica gagates* Latreille, *Lasius lasioides* (Emery), *Plagiolepis perperamus* Salata et al., *P. pygmaea* (Latreille), *Ponera coarctata* (Latreille), Solenopsis cf. fugax, *Tapinoma erraticum* (Latreille), *T. laconicus* Csősz et al., *Tetramorium kephalosi* Salata & Borowiec, T. cf. punctatum;

**Ionian Islands, Lefkada, Asprogerakata**: *Aphaenogaster balcanica* (Emery), *A. muelleriana* Wolf, *Camponotus aethiops* (Latreille), *C. dalmaticus* (Nylander), *Crematogaster schmidti* (Mayr), *Lepisiota frauenfeldi* (Mayr), Pheidole cf. pallidula, *Prenolepis nitens* (Mayr), *Tetramorium kephalosi* Salata & Borowiec; **Egklouvi**: *Aphaenogaster balcanica* (Emery), *Camponotus dalmaticus* (Nylander), *Cataglyphis nodus* (Brullé), *Crematogaster schmidti* (Mayr), *Messor wasmanni* Krausse, Pheidole cf. pallidula, *Temnothorax lichtensteini* (Bondroit), *T. rogeri* Emery;

**Ionian Islands, Zakynthos, 1 km E of Elies**: *Camponotus aethiops* (Latreille), *C. dalmaticus* (Nylander), *C. kiesenwetteri* (Roger), *C. lateralis* (Olivier), *C. oertzeni* Forel, *Crematogaster schmidti* (Mayr), *Messor wasmanni* Krausse, *Plagiolepis pygmaea* (Latreille), Temnothorax cf. tergestinus; **1 km N of Exo Chora**: *Aphaenogaster balcanica* (Emery), *Camponotus dalmaticus* (Nylander), *C. kiesenwetteri* (Roger), *C. oertzeni* Forel, *Crematogaster schmidti* (Mayr), *C. sordidula* (Nylander), *Lepisiota frauenfeldi* (Mayr), Pheidole cf. pallidula, *Plagiolepis pygmaea* (Latreille), *Temnothorax bulgaricus* (Forel), *T. rogeri* Emery, T. cf. tergestinus; **1.2 km N of Ano Vasilikos**: *Temnothorax exilis* (Emery); **1.2 km N of Vasilikos**: *Aphaenogaster balcanica* (Emery), *Camponotus aethiops* (Latreille), *C. dalmaticus* (Nylander), *C. kiesenwetteri* (Roger), *C. lateralis* (Olivier), *Colobopsis truncata* (Spinola), *Crematogaster schmidti* (Mayr), *C. sordidula* (Nylander), *Lepisiota frauenfeldi* (Mayr), *Plagiolepis pygmaea* (Latreille), *Temnothorax exilis* (Emery), *T. graecus* (Forel), *T. rogeri* Emery, T. cf. tergestinus; **1.2 km NE of Anafonitria**: *Aphaenogaster balcanica* (Emery), A. cf. epirotes, *Camponotus aethiops* (Latreille), *C. dalmaticus* (Nylander), *C. kiesenwetteri* (Roger), *Crematogaster schmidti* (Mayr), *Lepisiota frauenfeldi* (Mayr), Pheidole cf. pallidula, *Plagiolepis pygmaea* (Latreille), *Temnothorax exilis* (Emery), *T. muellerianus* (Finzi), T. cf. tergestinus; **1.2 km SW of Ano Vasilikos loc. 1**: *Aphaenogaster balcanica* (Emery), *Camponotus dalmaticus* (Nylander), *C. kiesenwetteri* (Roger), *C. lateralis* (Olivier), *C. oertzeni* Forel, *Crematogaster schmidti* (Mayr), *C. sordidula* (Nylander), *Lepisiota frauenfeldi* (Mayr), *Plagiolepis pygmaea* (Latreille), *Temnothorax exilis* (Emery); **1.2 km SW of Ano Vasilikos loc. 2**: *Aphaenogaster balcanica* (Emery), Aphaenogaster cf. epirotes, *Camponotus dalmaticus* (Nylander), *C. lateralis* (Olivier), *C. oertzeni* Forel, *Cataglyphis nodus* (Brullé), *Crematogaster schmidti* (Mayr), *C. sordidula* (Nylander), *Lepisiota melas* (Emery), *Messor wasmanni* Krausse, Pheidole cf. pallidula, *Plagiolepis pygmaea* (Latreille), *Temnothorax exilis* (Emery); **1.2 km SW of Skinaria**: *Aphaenogaster balcanica* (Emery), *Camponotus aethiops* (Latreille), *C. gestroi* Emery, *C. ionius* Emery, *C. kiesenwetteri* (Roger), *C. oertzeni* Forel, *Crematogaster sordidula* (Nylander), *Lepisiota melas* (Emery), *Messor wasmanni* Krausse, Pheidole cf. pallidula, *Plagiolepis pygmaea* (Latreille), *Temnothorax exilis* (Emery), *T. rogeri* Emery, T. cf. tergestinus, *Tetramorium diomedeum* Emery, *T. kephalosi* Salata & Borowiec; **1.4 km E of Ano Volimes**: *Aphaenogaster balcanica* (Emery), A. cf. epirotes, *A. muelleriana* Wolf, *Camponotus dalmaticus* (Nylander), *Crematogaster schmidti* (Mayr), *Lepisiota frauenfeldi* (Mayr), *Messor wasmanni* Krausse, Pheidole cf. pallidula, *Plagiolepis pygmaea* (Latreille), *Prenolepis nitens* (Mayr), *Temnothorax bulgaricus* (Forel), T. cf. tergestinus, *Tetramorium kephalosi* Salata & Borowiec; **1.5 km N of Exo Chora**: Aphaenogaster cf. epirotes, *Camponotus dalmaticus* (Nylander), *C. kiesenwetteri* (Roger), *C. lateralis* (Olivier), *C. oertzeni* Forel, *Crematogaster schmidti* (Mayr), *Lepisiota frauenfeldi* (Mayr), *Messor wasmanni* Krausse, *Plagiolepis pygmaea* (Latreille), *Temnothorax bulgaricus* (Forel), T. cf. tergestinus, *Tetramorium kephalosi* Salata & Borowiec; **1.7 km NE of Ano Volimes**: *Camponotus aethiops* (Latreille), *C. dalmaticus* (Nylander), *C. gestroi* Emery, *C. kiesenwetteri* (Roger), *C. lateralis* (Olivier), *Crematogaster schmidti* (Mayr), *C. sordidula* (Nylander), Pheidole cf. pallidula, Temnothorax cf. tergestinus; **1.8 km SW of Volimes**: *Aphaenogaster balcanica* (Emery), *Camponotus aethiops* (Latreille), *C. dalmaticus* (Nylander), *C. kiesenwetteri* (Roger), *Crematogaster schmidti* (Mayr), *C. sordidula* (Nylander), *Plagiolepis pygmaea* (Latreille), Temnothorax cf. tergestinus; **1.9 km W of Maries**: *Aphaenogaster balcanica* (Emery), A. cf. epirotes, *Camponotus aethiops* (Latreille), *C. dalmaticus* (Nylander), *C. kiesenwetteri* (Roger), *Crematogaster sordidula* (Nylander), *Lepisiota melas* (Emery), *Messor wasmanni* Krausse, Pheidole cf. pallidula, *Plagiolepis pygmaea* (Latreille), Temnothorax cf. tergestinus; **2.5 km NE of Maries**: *Aphaenogaster balcanica* (Emery), A. cf. epirotes, *A. subterraneoides* Emery, *Bothriomyrmex communista* Santschi, *Camponotus aethiops* (Latreille), *C. dalmaticus* (Nylander), *C. kiesenwetteri* (Roger), *Crematogaster schmidti* (Mayr), *C. sordidula* (Nylander), *Plagiolepis pygmaea* (Latreille), *Temnothorax rogeri* Emery, *Tetramorium kephalosi* Salata & Borowiec; **3.9 km NE of Maries**: *Camponotus aethiops* (Latreille), *C. dalmaticus* (Nylander), *Crematogaster schmidti* (Mayr), *Prenolepis nitens* (Mayr), Temnothorax cf. tergestinus, *Tetramorium diomedeum* Mayr; **470 m NE of Orthonies**: *Aphaenogaster balcanica* (Emery), *Camponotus aethiops* (Latreille), *C. dalmaticus* (Nylander), *C. kiesenwetteri* (Roger), *Crematogaster schmidti* (Mayr), *C. sordidula* (Nylander), *Lepisiota melas* (Emery), *Plagiolepis pygmaea* (Latreille), *Temnothorax bulgaricus* (Forel), *T. exilis* (Emery), Temnothorax cf. tergestinus; **500 m S of Apelati**: *Aphaenogaster balcanica* (Emery), *Camponotus dalmaticus* (Nylander), *C. lateralis* (Olivier), *Crematogaster ionia* Forel, *C. schmidti* (Mayr), *Messor wasmanni* Krausse, *Myrmecina graminicola* (Latreille), *Plagiolepis pygmaea* (Latreille), Temnothorax cf. tergestinus; **700 m SW of Koroni**: *Camponotus aethiops* (Latreille), *C. kiesenwetteri* (Roger), *C. lateralis* (Olivier), *Lepisiota melas* (Emery), Pheidole cf. pallidula, *Plagiolepis pygmaea* (Latreille), *Temnothorax exilis* (Emery), Temnothorax cf. tergestinus; **750 m S of Volimes**: *Camponotus dalmaticus* (Nylander), *C. gestroi* Emery, *C. lateralis* (Olivier), *Crematogaster schmidti* (Mayr), *C. sordidula* (Nylander), *Messor wasmanni* Krausse, *Plagiolepis pygmaea* (Latreille), *Temnothorax bulgaricus* (Forel), *T. rogeri* Emery, T. cf. tergestinus; **800 m SE of Xirokastello**: *Camponotus dalmaticus* (Nylander), *C. kiesenwetteri* (Roger), *C. lateralis* (Olivier), *C. oertzeni* Forel, *Colobopsis truncata* (Spinola), *Crematogaster schmidti* (Mayr), *Lepisiota melas* (Emery), *Messor wasmanni* Krausse, *Plagiolepis pygmaea* (Latreille), *Temnothorax exilis* (Emery), T. cf. tergestinus, Tetramorium cf. punctatum; **880 m S of Orthonies**: *Aphaenogaster balcanica* (Emery), A. cf. epirotes, *Camponotus aethiops* (Latreille), *C. dalmaticus* (Nylander), *C. kiesenwetteri* (Roger), *C. lateralis* (Olivier), *Crematogaster schmidti* (Mayr), *Lepisiota frauenfeldi* (Mayr), *L. melas* (Emery), Pheidole cf. pallidula, *Plagiolepis pygmaea* (Latreille), *Temnothorax bulgaricus* (Forel), *T. rogeri* Emery, T. cf. tergestinus; **Ag. Georgiou monastery**: *Camponotus aethiops* (Latreille), *C. dalmaticus* (Nylander), *Crematogaster schmidti* (Mayr), *Plagiolepis pygmaea* (Latreille); **Ag. Joannis**: *Camponotus aethiops* (Latreille), *C. kiesenwetteri* (Roger), *C. lateralis* (Olivier), *Crematogaster schmidti* (Mayr), *C. sordidula* (Nylander), Pheidole cf. pallidula, Solenopsis cf. lusitanica, *Temnothorax graecus* (Forel), *T. rogeri* Emery, T. cf. tergestinus, *Tetramorium kephalosi* Salata & Borowiec; **Argassi**: *Aphaenogaster balcanica* (Emery), *Camponotus dalmaticus* (Nylander), *C. kiesenwetteri* (Roger), *C. oertzeni* Forel, *Colobopsis truncata* (Spinola), *Crematogaster schmidti* (Mayr), *C. sordidula* (Nylander), *Lasius alienus* (Foerster), *Lepisiota melas* (Emery), *Messor ibericus* Santschi, *Monomorium monomorium* Bolton, *Nylanderia jaegerskioeldi* (Mayr), Pheidole cf. pallidula, *Plagiolepis perperamus* Salata et al., *P. pygmaea* (Latreille), *Tapinoma erraticum* (Latreille), *Temnothorax rogeri* Emery, *Tetramorium immigrans* Santschi, T. cf. punctatum; **Livia Mts. loc. 1**: *Aphaenogaster balcanica* (Emery), A. cf. epirotes, *Bothriomyrmex communista* Santschi, *Camponotus aethiops* (Latreille), *C. kiesenwetteri* (Roger), *Crematogaster schmidti* (Mayr), *C. sordidula* (Nylander), *Messor wasmanni* Krausse, *Prenolepis nitens* (Mayr), Tetramorium cf. punctatum; **Vrachionas Mts.**: *Aphaenogaster balcanica* (Emery), *Bothriomyrmex communista* Santschi, *Camponotus aethiops* (Latreille), *C. dalmaticus* (Nylander), *C. kiesenwetteri* (Roger), *C. oertzeni* Forel, *Crematogaster schmidti* (Mayr), *Messor wasmanni* Krausse, *Plagiolepis pygmaea* (Latreille), *Prenolepis nitens* (Mayr), Temnothorax cf. tergestinus, Tetramorium cf. punctatum; **s, W of Kampi**: *Aphaenogaster balcanica* (Emery), A. cf. epirotes, *A. muelleriana* Wolf, *Camponotus aethiops* (Latreille), *Crematogaster schmidti* (Mayr), Pheidole cf. pallidula, *Plagiolepis pygmaea* (Latreille), Solenopsis cf. lusitanica, *Temnothorax rogeri* Emery.

**Macedonia, Pieria, Platamonas Castle hill**: *Aphaenogaster epirotes* (Emery), *A. muelleriana* Wolf, A. cf. subterranea, *Camponotus dalmaticus* (Nylander), *C. lateralis* (Olivier), *C. piceus* (Leach), *Crematogaster ionia* Forel, *C. sordidula* (Nylander), *Lasius emarginatus* (Olivier), *L. turcicus* (Santschi), *Messor hellenius* Agosti & Collingwood, *Pheidole balcanica* Seifert, *Plagiolepis pygmaea* (Latreille), *Prenolepis nitens* (Mayr), *Temnothorax lichtensteini* (Bondroit); **road to P. Poroi loc. 1**: *Camponotus aethiops* (Latreille), *C. dalmaticus* (Nylander), *C. ionius* Emery, *C. piceus* (Leach), *Chalepoxenus muellerianus* (Finzi), *Crematogaster ionia* Forel, *Dolichoderus quadripunctatus* (Linnaeus), *Formica gagates* Latreille, *Lasius emarginatus* (Olivier), *Messor hellenius* Agosti & Collingwood, *Plagiolepis pygmaea* (Latreille), *Tapinoma erraticum* (Latreille), Temnothorax cf. exilis, *T. graecus* (Forel), *T. turcicus* (Santschi); **road to P. Poroi loc. 2**: *Camponotus aethiops* (Latreille), *C. dalmaticus* (Nylander), *C. piceus* (Leach), *Cataglyphis nodus* (Brullé), *Crematogaster schmidti* (Mayr), *Dolichoderus quadripunctatus* (Linnaeus); *Lasius emarginatus* (Olivier), *Lepisiota frauenfeldi* (Mayr), *Pheidole balcanica* Seifert, *Plagiolepis pygmaea* (Latreille), *Prenolepis nitens* (Mayr), *Tapinoma erraticum* (Latreille), *Temnothorax graecus* (Forel), *T. turcicus* (Santschi), *Tetramorium kephalosi* Salata & Borowiec; **road to P. Poroi loc. 3**: *Camponotus dalmaticus* (Nylander), *C. lateralis* (Olivier), *C. vagus* (Scopoli), *Crematogaster schmidti* (Mayr), *Dolichoderus quadripunctatus* (Linnaeus), *Lasius emarginatus* (Olivier), *Messor hellenius* Agosti & Collingwood, *Pheidole balcanica* Seifert, *Plagiolepis pygmaea* (Latreille), *Prenolepis nitens* (Mayr), *Temnothorax bulgaricus* (Forel), *T. morea* Csősz, Salata & Borowiec, *T. turcicus* (Santschi).

**Peloponnese, Korinthia, near Evrostina**: *Aphaenogaster subterranea* (Latreille), *Camponotus lateralis* (Olivier), *Crematogaster schmidti* (Mayr), *Dolichoderus quadripunctatus* (Linnaeus), *Lasius emarginatus* (Olivier), *L. turcicus* (Santschi), *Ponera coarctata* (Latreille), *Temnothorax laconicus* Csősz et al.

**Peloponnese, Laconia, Taygetos Mts., 1.5 km SW of Anavryti**: *Aphaenogaster balcanica* (Emery), *A. subterranea* (Latreille), *Camponotus dalmaticus* (Nylander), *C. oertzeni* Forel, *C. piceus* (Leach), *Cataglyphis nodus* (Brullé), *Crematogaster ionia* Forel, *Temnothorax laconicus* Csősz et al.

**Peloponnese, Messinia, Taygetos Mts., 0.7 km S of Dyrrachio**: *Camponotus lateralis* (Olivier), *Crematogaster schmidti* (Mayr), *Lasius flavus* (Fabricius), *L. illyricus* Zimmermann, *Temnothorax helenae* Csősz et al.; **Taygetos Mts., 0.8 km SE of Exochori**: *Aphaenogaster balcanica* (Emery), *Camponotus dalmaticus* (Nylander), *C. lateralis* (Olivier), *Crematogaster ionia* Forel, *Lepisiota frauenfeldi* (Mayr), *Messor structor* (Latreille), *M. wasmanni* Krausse, *Pheidole pallidula* (Nylander), *Plagiolepis pygmaea* (Latreille), Temnothorax cf. bulgaricus, *T. exilis* (Emery), *T. helenae* Csősz et al., *T. recedens* (Nylander), *Tetramorium kephalosi* Salata & Borowiec; **Taygetos Mts., 1.3 km S of Artemisia**: *Aphaenogaster subterranea* (Latreille), *Camponotus boghossiani* Forel, *Crematogaster schmidti* (Mayr), Lasius cf. alienus, *L. illyricus* Zimmermann, *Myrmecina graminicola* (Latreille), *Plagiolepis pygmaea* (Latreille), *Temnothorax crasecundus* Seifert & Csősz, *T. helenae* Csősz et al.; **Taygetos Mts., 1.8 km E of Saidona**: *Lasius illyricus* Zimmermann, *Plagiolepis pygmaea* (Latreille), *Temnothorax crasecundus* Seifert & Csősz, Tetramorium cf. caespitum; **Taygetos Mts., 2 km W of Arachova**: Aphaenogaster cf. muelleriana, *Bothriomyrmex communista* Santschi, *Camponotus boghossiani* Forel, *C. dalmaticus* (Nylander), *C. laconicus* Emery, *Cataglyphis nodus* (Brullé), *Crematogaster schmidti* (Mayr), *Lepisiota frauenfeldi* (Mayr), *Messor wasmanni* Krausse, *Pheidole pallidula* (Nylander), *Plagiolepis pygmaea* (Latreille), *Stigmatomma denticulatum* Roger, *Tapinoma erraticum* (Latreille), Temnothorax cf. bulgaricus, *T. laconicus* Csősz et al., T. cf. luteus, *T. rogeri* Emery, *Tetramorium kephalosi* Salata & Borowiec, Tetramorium cf. punctatum; **Taygetos Mts., Arachova**: *Camponotus aethiops* (Latreille), *C. laconicus* Emery, *C. piceus* (Leach), *Cataglyphis nodus* (Brullé), *Crematogaster schmidti* (Mayr), *Dolichoderus quadripunctatus* (Linnaeus), Lasius cf. alienus, *L. illyricus* Zimmermann, *Lepisiota frauenfeldi* (Mayr), *Messor wasmanni* Krausse, *Pheidole pallidula* (Nylander), *Plagiolepis pygmaea* (Latreille), *Tapinoma erraticum* (Latreille), Temnothorax cf. bulgaricus, *T. parvulus* (Schenck); **Taygetos Mts., Chora Getson**: *Aphaenogaster balcanica* (Emery), A. cf. muelleriana, *Camponotus boghossiani* Forel, *Crematogaster schmidti* (Mayr), *Dolichoderus quadripunctatus* (Linnaeus), Lasius cf. alienus, *L. illyricus* Zimmermann, *Lepisiota frauenfeldi* (Mayr), *Pheidole pallidula* (Nylander), *Plagiolepis pygmaea* (Latreille), Temnothorax cf. bulgaricus, *T. laconicus* Csősz et al., *T. recedens* (Nylander), *T. strymonensis* Csősz, Salata & Borowiec; **Taygetos Mts., Karveli**: Aphaenogaster cf. muelleriana, *Crematogaster schmidti* (Mayr), Lasius cf. alienus, *L. illyricus* Zimmermann, *Myrmecina graminicola* (Latreille), *Temnothorax helenae* Csősz et al., *T. strymonensis* Csősz, Salata & Borowiec; **Tetrazi Mts., 0.5 km E of Vastas**: *Aphaenogaster balcanica* (Emery), *Camponotus dalmaticus* (Nylander), *C. fallax* (Nylander), *C. laconicus* Csősz et al., *C. piceus* (Leach), *Colobopsis truncata* (Spinola), *Crematogaster schmidti* (Mayr), *Formica gagates* Latreille, *Lasius illyricus* Zimmermann, *Pheidole pallidula* (Nylander), *Plagiolepis pygmaea* (Latreille), *Temnothorax helenae* Csősz et al., *T. laconicus* Csősz et al., *T. morea* Csősz, Salata & Borowiec, *T. rogeri* Emery; **Tetrazi Mts., Isaris**: *Aphaenogaster balcanica* (Emery), *A. subterranea* (Latreille), *Camponotus dalmaticus* (Nylander), *C. laconicus* Csősz et al., *C. lateralis* (Olivier), *Crematogaster schmidti* (Mayr), *Dolichoderus quadripunctatus* (Linnaeus), Lasius cf. alienus, *L. illyricus* Zimmermann, *Lepisiota frauenfeldi* (Mayr), Temnothorax cf. bulgaricus, *T. helenae* Csősz et al.,; **Tetrazi Mts., Karnasi**: *Aphaenogaster balcanica* (Emery), A. cf. muelleriana, *Camponotus dalmaticus* (Nylander), *C. lateralis* (Olivier), *Crematogaster schmidti* (Mayr), *Lasius illyricus* Zimmermann, *Messor hellenius* Agosti & Collingwood, *Pheidole pallidula* (Nylander), Temnothorax cf. bulgaricus, *Temnothorax laconicus* Csősz et al., *T. morea* Csősz, Salata & Borowiec, *T. rogeri* Emery, *Tetramorium kephalosi* Salata & Borowiec.

**Sterea Ellas, Aetolia-Acarnania, Psila Alonia**: *Aphaenogaster balcanica* (Emery), A. cf. muelleriana, *A. subterranea* (Latreille), *Camponotus dalmaticus* (Nylander), *C. lateralis* (Olivier), *Cataglyphis nodus* (Brullé), *Crematogaster schmidti* (Mayr), *Dolichoderus quadripunctatus* (Linnaeus), *Lasius illyricus* Zimmermann, *Messor wasmanni* Krausse, Pheidole cf. pallidula, *Plagiolepis pygmaea* (Latreille), Solenopsis cf. lusitanica, *Temnothorax crassispinus* (Karavaiev).

**Sterea Ellas, Euboea, 1.2 km NW of Gerontas**: *Aphaenogaster balcanica* (Emery), *Camponotus aethiops* (Latreille), *C. dalmaticus* (Nylander), *C. gestroi* Emery, *C. kiesenwetteri* (Roger), *C. laconicus* Emery, *C. lateralis* (Olivier), *C. piceus* (Leach), *Cataglyphis aenescens* (Nylander), *Crematogaster ionia* Forel, *C. schmidti* (Mayr), Pheidole cf. pallidula, *Plagiolepis pygmaea* (Latreille), *Tapinoma erraticum* (Latreille), *Temnothorax graecus* (Forel), *T. recedens* (Nylander); **2.3 km S of Stropones**: *Camponotus aethiops* (Latreille), *C. piceus* (Leach), *C. vagus* (Scopoli), *Cataglyphis nodus* (Brullé), *Formica fusca* Karavaiev, *Lasius alienus* Förster, *L. flavus* (Fabricius), *Pheidole pallidula* (Nylander), *Temnothorax crasecundus* Seifert & Csősz, *T. helenae* Csősz et al., *T. unifasciatus* (Latreille); **300 m NW of Agios**: *Camponotus aethiops* (Latreille), *C. dalmaticus* (Nylander), *Cataglyphis nodus* (Brullé), *Crematogaster ionia* Forel, *Dolichoderus quadripunctatus* (Linnaeus), *Formica fusca* Karavaiev, *Lasius lasioides* (Emery), Pheidole cf. pallidula, *Plagiolepis pygmaea* (Latreille), *Temnothorax lichtensteini* (Bondroit), *T. parvulus* (Schenck), *T. recedens* (Nylander), *T. unifasciatus* (Latreille); **570 m NW of Drosia**: *Aphaenogaster balcanica* (Emery), *A. subterranea* (Latreille), *Camponotus dalmaticus* (Nylander), *C. ionius* Emery, *C. lateralis* (Olivier), *C. piceus* (Leach), *C. vagus* (Scopoli), *Cataglyphis nodus* (Brullé), *Crematogaster ionia* Forel, *C. schmidti* (Mayr), *Lepisiota frauenfeldi* (Mayr), *Messor hellenius* Agosti & Collingwood, *Temnothorax bulgaricus* (Forel).

**Thessaly, Larissa, Mt. Olympus, 5.3 km E of Olympiada**: *Aphaenogaster subterranea* (Latreille), *Camponotus aethiops* (Latreille), *C. lateralis* (Olivier), *C. piceus* (Leach), *Crematogaster schmidti* (Mayr), *Dolichoderus quadripunctatus* (Linnaeus), *Formica cunicularia* Latreille, *F. gagates* Latreille, *Lasius alienus* Förster, *L. emarginatus* (Olivier), *Messor hellenius* Agosti & Collingwood, *Pheidole pallidula* (Nylander), *Plagiolepis pygmaea* (Latreille), *Prenolepis nitens* (Mayr), Solenopsis cf. lusitanica, *Tapinoma erraticum* (Latreille), *Temnothorax recedens* (Nylander), *Tetramorium kephalosi* Salata & Borowiec, *Tetramorium moravicum* Kratochvil; **Mt. Ossa, 2.4 km SE of Karitsa**: *Aphaenogaster subterranea* (Latreille), *Camponotus lateralis* (Olivier), *Cataglyphis nodus* (Brullé), *Crematogaster schmidti* (Mayr), *Dolichoderus quadripunctatus* (Linnaeus), *Formica gagates* Latreille, *Lasius emarginatus* (Olivier), *Myrmoxenus gordiagini* Ruzsky, *Plagiolepis pygmaea* (Latreille), *Temnothorax crasecundus* Seifert & Csősz, T. cf. subtilis-*helenae*, *T. tauricus* (Ruzsky), T. cf. unifasciatus, *Tetramorium moravicum* Kratochvil; **Mt. Ossa, 600 m SE of Karitsa**: *Aphaenogaster subterranea* (Latreille), *Camponotus fallax* (Nylander), *Colobopsis truncata* (Spinola), *Lasius emarginatus* (Olivier), *Plagiolepis pygmaea* (Latreille), *Prenolepis nitens* (Mayr), *Temnothorax crasecundus* Seifert & Csősz, *T. lichtensteini* (Bondroit), T. cf. subtilis-helenae, T. cf. unifasciatus; **Mt. Ossa, Kokkino Nero**: *Aphaenogaster subterranea* (Latreille), *Camponotus aethiops* (Latreille), *C. dalmaticus* (Nylander), *C. lateralis* (Olivier), *Colobopsis truncata* (Spinola), *Crematogaster schmidti* (Mayr), *Dolichoderus quadripunctatus* (Linnaeus), *Formica cunicularia* Latreille, *Lasius alienus* Förster, *L. emarginatus* (Olivier), *Messor hellenius* Agosti & Collingwood, *Pheidole pallidula* (Nylander), *Prenolepis nitens* (Mayr), *Temnothorax tauricus* (Ruzsky), T. cf. unifasciatus, Tetramorium cf. caespitum.

#### Temnothorax messiniaensis sp. nov.

Taxon classificationAnimaliaHymenopteraFormicidae

DA472519-49D9-5486-A251-10DB0177D8A5

http://zoobank.org/0C110D03-C293-4AD1-B179-3A1F53E7491E

**Figures 3, 4.**
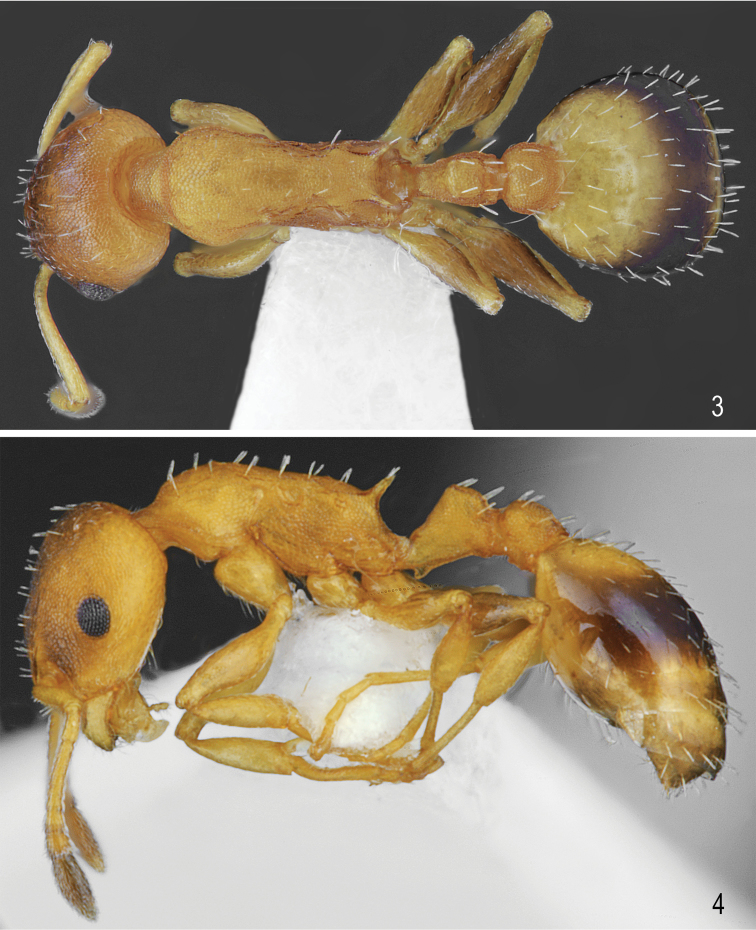
Worker of *Temnothorax messiniaensis* sp. nov. **3** Dorsal **4** Lateral.

**Figures 5, 6.**
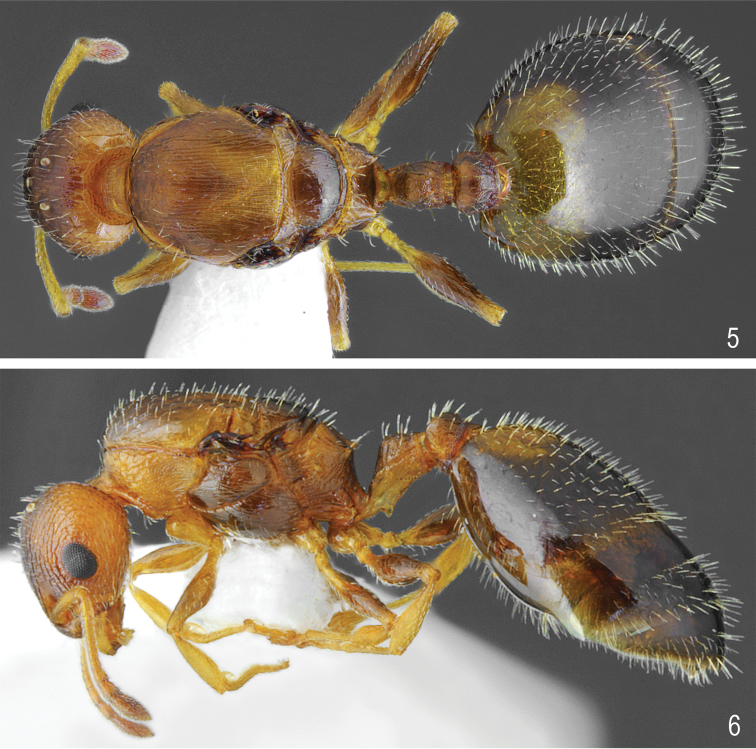
Gyne of *Temnothorax messiniaensis* sp. nov. **5** Dorsal **6** Lateral.

Figs 3–4 , 5–6 , 10 , 12 , 14

##### Type material.

**Holotype**, worker (pin) (CASENT0846796): GREECE, Pel., Messinia | 2 km E of Kalamata, 65 m, | 37.01863N / 22.15626E | 12 VI 2016, L. Borowiec || Collection L. Borowiec | Formicidae | LBC-GR01997 (MNHW).

**Paratypes**: • 3Q., 7w. (pin) (CASENT0846639-CASENT0846648): the same nest sample as holotype (DBET, BMNH, CASC, MHNG); • 1w. (pin) (CASENT0846649): GREECE, Pel., Messinia | 1.4 km S of Flesiada, 700 m | 37.08964N, 21.76581E | 16 VI 2016, L. Borowiec || Collection L. Borowiec | Formicidae | LBC-GR02091 (DBET); • 1w. (pin) (CASENT0846650): GREECE, Pel., Messinia | Kalamata, old centre, 60 m | 37.04617N, 22.11691E | 11 VI 2016, L. Borowiec || Collection L. Borowiec | Formicidae | LBC-GR02216 (DBET); • 2w. (pin) (CASENT0846651-CASENT0846652): GREECE, Pel., Messinia | Kalamata, railway park, 8m | 37.03157N, 22.11004E | 11 VI 2016, L. Borowiec || Collection L. Borowiec | Formicidae | LBC-GR01989 (DBET); • 2w. (pin) (CASENT0846653-CASENT0846654): GREECE, Pel., Messinia | 0.8 km N of Koromilea, 485 m | 37.16272N, 21.84809E | 16 VI 2016, L. Borowiec || Collection L. Borowiec | Formicidae | LBC-GR02103 (DBET); • 1w. (pin) (CASENT0846655): GREECE, Pel., Messinia | 0.8 km SE of Exochori, 535 m | 36.89582N, 22.27464E | 20 VI 2016, L. Borowiec || Collection L. Borowiec | Formicidae | LBC-GR02658 (DBET); • 1w. (pin) (CASENT0846656): GREECE, Pel., Messinia | 0.8 km W of Eleochori, 481 m 37.03838N, 22.17227E | 13 VI 2016, lL. Borowiec || Collection L. Borowiec | Formicidae | LBC-GR02005 (DBET).

##### Other material.

**Greece. Ionian Islands, Cephalonia**: • 7w. (EtOH): Avithos Lake, shrubs around small lake, 38.17203N/ 20.71107E, 288 m, 2019-06-10, leg. L. Borowiec; • 3w. (pin), 9w. (EtOH): 1.6 km SW of Digaleto, small gorge with oaks, 38.16558N, 20.67099E, 564 m, 2019-06-11, leg. L. Borowiec; • 1w. (EtOH): 1.8 km SW of Digaleto, pastures with oaks, 38.16593N, 20.66788E, 580 m, 2019-06-11, leg. L. Borowiec; • 7w. (EtOH): Kapandriti vicinity, roadsides with herbs, 38.12913N/20.72447E, 320 m, 2019-06-09, leg. L. Borowiec; • 12w. (EtOH): Katapodata; roadsides with shrubs, 38.23337N/20.64594E, 100 m, 2019-06-10, leg. L. Borowiec; • 1w. (pin) (CASENT0846797), 43w. (EtOH): 800 m S of Kateleios, roadsides with bushes, 38.07066N/20.75329E, 20 m, 2019-06-09, leg. L. Borowiec; • 5w. (EtOH): 1.5 km NE of Koulourata, mixed forest on shrubs, 38.20667N/ 20.67715E, 273 m, 2019-06-10, leg. L. Borowiec; • 48w. (EtOH): Kremmidi, roadsides with bushes, 38.09048N/20,74471E, 285 m, 2019-06-09, leg. L. Borowiec; • 7w. (EtOH): Moni Aprilion, hill with oak forest and rocks, 38.26221N/20.66651E, 220 m, 2019-06-10, leg. L. Borowiec; • 48w. (EtOH): 1 km NW of Pastra, roadsides with bushes, 38.10058N/20.7421E, 300 m, 2019-06-09, leg. L. Borowiec; • 4w. (EtOH): 1.7 km NW of Pastra, pastures, on shrubs, 38.1084N/20.74085E, 300 m, 2019-06-09, leg. L. Borowiec; • 6w. (pin) (CASENT0846798-CASENT0846803): n. Peratata, pine forest on a rocky hill, 38.14058N/20.55038E, 211 m, 2014-06-24, leg. L. Borowiec; • 11w. (EtOH): ancient Same; roadsides with shrubs, 38.2522N/20.66423E, 220 m, 2019-06-10, leg. L. Borowiec; • 3w. (pin) (CASENT0846804-CASENT0846806), 6w. (EtOH): Skala vicinity loc. 1, small gorge with mediterranean shrubs, 38.08178N/20.79275E, 40 m, 2019-06-07, leg. L. Borowiec; • 5w. (pin) (CASENT0846807-CASENT0846811), 44w, 1q (EtOH): Skala vicinity loc. 2, small gorge with mediterranean shrubs, nest inside dry branch of shrub, 38.08221N, 20.79504E, 34 m, 2019-06-07, leg. L. Borowiec; • 61w. (EtOH): rd. Skala-Poros; mediterranean shrubs, 38.12872N/20.79576E, 5 m, 2019-06-12, leg. L. Borowiec. **Ionian Islands, Zakynthos**: • 3w. (pin) (CASENT0846812-CASENT0846814), 3w. (EtOH): 1 km N of Exo Chora, mixed forest, 37.81063N/20.68459E, 430 m, 2018-05-08, leg. L. Borowiec; • 1w. (pin) (CASENT0846815): 1.2 km N of Vasilikos, roadsides along olive plantation and pasture with oak shrubs, 37.72456N/20.97786E, 30 m, 2018-05-05, leg. L. Borowiec; • 5w. (pin) (CASENT0846816-CASENT0846820), 1w. (EtOH): 1.2 km NE of Anafonitria, shrubs around burned forests, 37.85489N/20.64124E, 475 m, 2018-05-10, leg. L. Borowiec; • 1w. (pin) (CASENT0846821): 1.2 km SE of Loucha, roadsides in cypress forest, 37.78617N/ 20.73706E, 445 m, 2018-05-09, leg. L. Borowiec; • 5w. (pin), 3w. (EtOH): 1.2 km SW of Skinaria, limestone hills after burned forests, 37.87694N/20.69272E, 375 m, 2018-05-06, leg. L. Borowiec; • 2w. (pin) (CASENT0846822-CASENT0846823): 1.4 km S of Lithakia, shrubs along olive plantation, 37.70641N/20.82342E, 225 m, 2018-05-07, leg. L. Borowiec; • 2w. (pin) (CASENT0846824-CASENT0846825): 1.8 km SW of Volimes, shrubs along roadsides, 37.86472N/20.64234E, 350 m, 2018-05-10, leg. L. Borowiec; • 3w. (pin) (CASENT0846826-CASENT0846827), 40w. (EtOH): 1.9 km W of Maries, roadsides in burned forests, 37.818N/20.65556E, 290 m, 2018-05-09, leg. L. Borowiec; • 3w. (pin) (CASENT0846828-CASENT0846830), 19w. (EtOH): 330 m S of Stimies, shrubs around olive plantation, 37.69009N/20.79988E, 245 m, 2018-05-07, leg. L. Borowiec; • 1w. (pin) (CASENT0846831): 470 m NE of Orthonies, shrubs along roadsides, 37.85435N/ 20.69843E, 405 m, 2018-05-10, leg. L. Borowiec; 1w. (pin) (CASENT0846832): 580 m SW of Lithakia, shrubs along roadsides, 37.71491N/20.8242E, 225 m, 2018-05-07, leg. L. Borowiec; • 3w. (pin) (CASENT0846833-CASENT0846835), 4w. (EtOH): 600 m E of Ag. Leon, shrubs in pine forest, 37.77045N/20.72959E, 600 m, 2018-05-09, leg. L. Borowiec; • 2w. (pin) (CASENT0846836-CASENT0846837), 3w. (EtOH): 700 m SW of Koroni, maquis, 37.86582N/20.71753E, 290 m, 2018-05-10, leg. L. Borowiec; • 2w. (pin) (CASENT0846838-CASENT0846839): 800 m SE of Xirokastello, roadsides along olive plantation, 37.73491N/20.95139E, 75 m, 2018-05-05, leg. L. Borowiec; • 3w. (pin) (CASENT0846840-CASENT0846842), 9w. (EtOH): 880 m S of Orthonies, shrubs in cypress forest, 37.84462N/ 20.69843E, 390 m, 2018-05-10, leg. L. Borowiec; • 3w. (pin) (CASENT0846843-CASENT0846845), 2w. (EtOH): Ag. Georgiou monastery, shrubs along roadsides, 37.85971N/20.63646E, 330 m, 2018-05-10, leg. L. Borowiec; • 2w. (pin) (CASENT0846846-CASENT0846847): Ag. Joannis, roadsides with shrubs, 37.72924N/20.94553E, 165 m, 2018-05-05, leg. L. Borowiec; • 1w. (pin) (CASENT0846848): Vrachionas Mts., mountain pastures with shrubs, 37.81798N/20.70621E, 670 m, 2018-05-08, leg. L. Borowiec; **Peloponnese, Achaia**: • 1w (pin) (CASENT0846849): Kalavrita, 710 m, 38.03342N, 22.10456E, leg. C. Lebas; **Peloponnese, Lakonia**: • 1w (pin) (CASENT0846850): Mistra vicinity, 378 m, 37.08115N, 22.36545E, leg. C. Lebas.

##### Terra typica.

Greece, Peloponnese, Messinia.

##### Differential diagnosis.

Both *T. messiniaensis* sp. nov. and *T. turcicus* (Santschi) are characterised by very long propodeal spines, character strongly distinguishing them from *T. brackoi* sp. nov. They differ from species of the *T. interruptus* group in lack of wide frontal lobes and not distinctly triangular propodeal spines; from *T. affinis* they differ in brighter body colouration and shape of propodeal spines (*T. affinis* has propodeal spines thin and never triangular, while *T. messiniaensis* and *T. turcicus* have propodeal spines more triangular, with wider base); from *T. kemali* both new species differ in absence of distinctly arched dorsum of petiole node and more triangular shape of propodeal spines, additionally *T. messiniaensis* differs from *T. kemali* in not reduced sculpture on frons centre and *T. turcicus* differs from *T. kemali* in very thin dark band on posterior part of first gastral tergite. *T. messiniaensis* differs from *T. turcicus* in not reduced sculpture on frons centre and wider dark band on posterior part of first gastral tergite. From specimens of *T. aveli* with long propodeal spines and almost complete microreticulation of head *T. messiniaensis* differs in less convex mesosoma, longer head, usually darkened gena and propodeal spines directed more upwards.

**Description of worker** (n = 10): HL: 0.619 ± 0.04 (0.565–0.682); HW: 0.515 ± 0.03 (0.471–0.564); SL: 0.428 ± 0.02 (0.388–0.459); EL: 0.134 ± 0.008 (0.129–0.153); EW: 0.102 ± 0.01 (0.094–0.118); WL: 0.723 ± 0.05 (0.624–0.765); PSL: 0.171 ± 0.02 (0.135–0.188); SDL: 0.109 ± 0.02 (0.082–0.159); PEL: 0.263 ± 0.02 (0.224–0.282); PPL: 0.167 ± 0.007 (0.152–0.176); PEH: 0.207 ± 0.009 (0.188–0.281); PPH: 0.195 ± 0.01 (0.176–0.212); PNW: 0.359 ± 0.03 (0.318–0.400); PLW: 0.162 ± 0.01 (0.141–0.176); PPW: 0.208 ± 0.01 (0.188–0.223); CI: 83.4 ± 2.5 (78.9–86.6); SI1: 69.1 ± 2.3 (65.5–72.5); SI2: 82.9 ± 2.4 (78.6–87.6); MI: 49.7 ± 1.6 (47.6–52.3); EI1: 76.4 ± 6.8 (66.7–90.9); EI2: 16.5 ± 1.1 (14.5–18.2); PI: 127.2 ± 4.5 (118.0–133.3); PPI: 85.9 ± 4.1 (77.7–93.3); PSI: 170.8 ± 16.8 (153.8–200.0).

##### Colour.

Head, antennae, mesosoma, petiole, postpetiole and legs uniformly yellow to ochre, often posterior part of frons, gena and femora partly darkened, occasionally also antennal club slightly darkened. Gaster yellow, only the first gaster tergite with wide, dark band on its posterior part (Figs 3, 4). **Head.** Rectangular, but slightly longer than in both Greek congeners, 1.2 times as long as wide, lateral surfaces below and above eyes gently convex, posterior edges convex, occipital margin of head straight or slightly concave (Figs 10, 14). Anterior margin of the clypeus slightly convex, medial notch absent. Eyes moderate, oval, 1.31 times as long as wide. Antennal scape short, in lateral view slightly curved, 0.69 times as long as length of the head, in apex gradually widened, its base with small, triangular tooth, funiculus long, club 3-segmented (Fig. 10). Surface of scape with very fine microreticulation, shiny, covered with thin, moderate dense, decumbent setae. Mandibles rounded with thick sparse, longitudinal striae, shiny. Clypeus shiny with thick, sparse, longitudinal striae, area between striae smooth and shiny. Frontal carinae short, not extending beyond frontal lobes. Antennal fossa deep, with irregular, dense rugosity and sometimes with a few thin, roundly curved striae. Frontal lobes narrow, smooth with slight, dense longitudinal striation (Fig. 14). Frons, vertex and temples with dense, thick, reticulation, central surface of frons with longitudinal reticulation and sometimes with additional thin, longitudinal striation, striae sometimes interrupted, surface between striation smooth; malar area with irregular, thick, reticulation, space between reticulation smooth or with very sparse microreticulation, shiny; genae with sparser, than on frons, and thick reticulation, shiny. Frons and vertex with erect, pale, short and thick setae (Fig. 14). **Mesosoma.** Elongate, approximately twice as long as wide, slightly arched in profile. Metanotal groove absent or slightly marked as in the species from the *Temnothorax angustulus* group. Pronotum convex on sides. Propodeal spines long, directed upward, with base slightly to moderate wider than apex, tips sharp (Fig. 4). Whole surface with dense, reticulation, sometimes its dorsal surface and lateral surfaces of pronotum and mesonotum with additional thick, sparse to moderate dense longitudinal striation or longitudinal reticulation. Area between thick sculpture shiny, smooth or sometimes with sparse, fine microreticulation (Fig. 4). Entire mesosoma bearing erect, pale, short and thick setae (Figs 3, 4). **Petiole.** In lateral view, with short peduncle, node moderate high, with anterior face flat, and posterior face convex and dorsum flat or slightly convex. Peduncle and petiolar node shiny, with thick, dense reticulation and sometimes thick, sparse longitudinal wrinkles, area between rugae smooth, dorsum with sparser reticulation. Dorsal surface bearing sparse, short, erect setae (Fig. 4). **Postpetiole.** In lateral view, regularly convex, apical half with gently convex sides (Figs 3, 4), on the whole surface shiny, with thick, dense reticulation, dorsum with sparser reticulation; area between rugae smooth. Dorsal surface bearing sparse, short, erect setae (Figs 3, 4). **Gaster.** Gaster smooth and shiny, bearing erect, thin, pale setae (Figs 3, 4).

**Description of gyne** (n = 3): **Colour.** Head in frontal part yellowish brown to brown, gena and temples yellowish, border between dark and pale parts of head diffused. Antennae uniformly yellow. Pronotum yellow, scutum yellow laterally and yellowish brown anteriorly, without distinct borders between darker and paler parts. Mesosterna brown, propodeum yellowish dorsally and gradually darker ventrally, petiole and postpetiole brown dorsally and yellow ventrally. Legs mostly yellow, mid- and hind femora largely brown centrally. Gastral tergites mostly brown, first tergite with large yellowish spot basally, all tergites with yellowish to yellowish brown posterior margin (Figs 5, 6). **Head.** Eyes big, oval [EL / HW: 0.30]. Antennal scape short [SL / HW: 42], not reaching occipital margin of head. Clypeus shiny with distinct, longitudinally carinulae, interstices smooth. Antennal fossa deep, with concentric carinae, interspaces smooth. Frontal lobes moderately wide 0.4 times as wide as head width, microreticulate with thick longitudinal costae. Frons shiny, entire surface longitudinally costate, interstices on sides distinctly microreticulate, in central part microreticulation diffused and surface appears partly smooth and shiny. Area above eyes and sides of head microreticulate and longitudinally costate, interstices appear slightly rugulose. Entire head bearing suberect to erect, pale and thin setae. **Mesosoma.** Pronotum anteriorly with regular microreticulation and on sides microreticulate with thin longitudinal costae. Scutum with dense, regular, thin longitudinal costae and more or less diffused microreticulation between costae, appears slightly shiny. Scutellum mostly with thin longitudinal costae only marrow median part with diffused sculpture, appears shiny (Fig. 5). Metanotum with slight sculpture, rugulose or punctate. Propodeum with distinct sculpture. Propodeal spines medium length [PSL / HW: 0.33], wide at base, triangular, straight, with acute apex. Area above propodeal spines with transverse, thin costae, dorsal surface of spines with longitudinal costae, sides of propodeum with concentric costae only area close to base of spines distinctly mocroreticulate but without costae, interstices between costae distinctly microreticulate, shiny. Area between propodeal spines on sides with longitudinal costae, centrally with distinct microreticulation, area below spines with transverse costae. Anepisternum and katepisternum with longitudinal costae and microreticulated interspaces, only anterior and posterior corners of anepisternum with small smooth and shiny areas, and katepisternum close to ventral margin with diffused costae. Metaepisternum and metakatepisternum, with dense, longitudinal costae and microreticulate interspaces only metakatepisternum close to ventral margin with diffused microreticulation and partly shiny. Dorsal surface of mesosoma with sparse, erect, long, thick and pale setae (Figs 5, 6). **Petiole and postpetiole.** Shiny anteriorly, dorsal and lateral surface microreticulate with sparse longitudinally costae. **Gaster.** Smooth and shiny, first tergite on whole surface and rest of tergites posteriorly bearing moderately dense, long, erect setae and sparse, short adhering setae (Figs 5, 6).

##### Etymology.

Named after the historical Greek land of Messinia (Μεσσηνία), Peloponnese, where specimens from the type series were collected.

##### General distribution.

Greece: southern Ionian Islands and Peloponnese.

##### Biology.

Specimens of *T. messiniaensis* were collected from sunny localities in lowlands and highlands (8 - 670 m a.s.l). The species was noted in various habitats, most often on shrubs growing along roadsides and olive plantations, maquis, phrygana and forests (cypress, deciduous and mixed). We noted also its presence in a park in a centre of a city and, occasionally, in shrubs located in a pine forest located on a rocky hill. Nests were found inside dry stems of various herbs and shrubs. Colonies polygynous.

The following ant species were recorded in the same areas as *Temnothorax messiniaensis*:

For localities on Ionian Islands: Cephalonia: Avithos Lake, Katapodata, 1.5 NE of Koulourata ancient Same, rd. Skala-Poros, Zakynthos: 1 km N of Exo Chora, 1.2 km N of Vasilikos, 1.2 km NE of Anafonitria, 1.2 km SW of Skinaria, 1.8 km SW of Volimes, 1.9 km W of Maries, 470 m NE of Orthonies, 700 m SW of Koroni, 800 m SE of Xirokastello, 880 m S of Orthonies, Ag. Georgiou monastery, Ag. Joannis, Vrachionas Mts. and Peloponnese, Messinia, Taygetos Mts., 0.8 km SE of Exochori see *Temnothorax brackoi*.

**Ionian Islands, Cephalonia, 1.6 km SW of Digaleto**: *Aphaenogaster balcanica* (Emery), *A. muelleriana* Wolf, *Camponotus aethiops* (Latreille), *C. dalmaticus* (Nylander), *Crematogaster schmidti* (Mayr), *Pheidole balcanica* Seifert, *Plagiolepis pygmaea* (Latreille), *Temnothorax bulgaricus* (Forel), *T. laconicus* Csősz et al., *T. rogeri* Emery, *T. strymonensis* Csősz, Salata & Borowiec; *Tetramorium kephalosi* Salata & Borowiec; **1.8 km SW of Digaleto**: *Aphaenogaster balcanica* (Emery), *Camponotus aethiops* (Latreille), *C. dalmaticus* (Nylander), *Crematogaster sordidula* (Nylander), *Pheidole balcanica* Seifert, *Plagiolepis pygmaea* (Latreille), *Temnothorax laconicus* Csősz et al., *T. rogeri* Emery; **Kapandriti vicinity**: *Aphaenogaster balcanica* (Emery), *Camponotus dalmaticus* (Nylander), *C. gestroi* Forel, *C. kiesenwetteri* (Roger), *Colobopsis truncata* (Spinola), *Crematogaster schmidti* (Mayr), *Plagiolepis pygmaea* (Latreille), *Temnothorax leviceps* (Emery); **800 m S of Kateleios**: *Camponotus dalmaticus* (Nylander), *C. lateralis* (Olivier), *Crematogaster schmidti* (Mayr), *C. sordidula* (Nylander), *Lasius alienus* (Förster), *L. illyricus* Zimmermann, *Liometopum microcephalum* (Panzer), *Pheidole balcanica* Seifert, *Plagiolepis pygmaea* (Latreille), *Temnothorax graecus* (Forel), *T. leviceps* (Emery), T. cf. unifasciatus; **Kremmidi**: *Aphaenogaster balcanica* (Emery), *Camponotus aethiops* (Latreille), *C. gestroi* Forel, *C. dalmaticus* (Nylander), *C. kiesenwetteri* (Roger), *Crematogaster schmidti* (Mayr), *C. sordidula* (Nylander), *Pheidole balcanica* Seifert; *Plagiolepis pygmaea* (Latreille), *Temnothorax bulgaricus* (Forel), *T. graecus* (Forel), *T. leviceps* (Emery); **Moni Aprilion**: *Aphaenogaster balcanica* (Emery), *Camponotus kiesenwetteri* (Roger), *Pheidole balcanica* Seifert, *Plagiolepis pygmaea* (Latreille), *Temnothorax rogeri* Emery, *T. strymonensis* Csősz, Salata & Borowiec; **1.7 km NW of Pastra**: *Aphaenogaster balcanica* (Emery), A. cf. epirotes, *Bothriomyrmex communistus* Santschi, *Camponotus dalmaticus* (Nylander), *C. gestroi* Forel, *C. kiesenwetteri* (Roger), *C. lateralis* (Olivier), *Crematogaster schmidti* (Mayr), *C. sordidula* (Nylander), *Lepisiota frauenfeldi* (Mayr), *Messor wasmanni* Krausse, *Plagiolepis pygmaea* (Latreille), *Tetramorium kephalosi* Salata & Borowiec; **near Peratata**: *Aphaenogaster balcanica* (Emery), *A. muelleriana* Wolf, *Camponotus aethiops* (Latreille), *C. dalmaticus* (Nylander), *C. lateralis* (Olivier), *Crematogaster schmidti* (Mayr), *C. sordidula* (Nylander), *Lasius lasioides* (Emery), *Messor ibericus* Santschi, *Pheidole pallidula* (Nylander), *Plagiolepis pygmaea* (Latreille), *Temnothorax clypeatus* (Mayr); **Skala vicinity loc. 1**: *Aphaenogaster balcanica* (Emery), *Camponotus aethiops* (Latreille), *C. dalmaticus* (Nylander), *C. kiesenwetteri* (Roger), *Colobopsis truncata* (Spinola), *Crematogaster sordidula* (Nylander), *Plagiolepis pallescens* Forel, *P. pygmaea* (Latreille), *Proformica oculatissima* (Forel) Temnothorax cf. exilis, *Tetramorium kephalosi* Salata & Borowiec; **Skala vicinity loc. 2**: *Aphaenogaster balcanica* (Emery), *Camponotus dalmaticus* (Nylander), *C. kiesenwetteri* (Roger), *C. lateralis* (Olivier), *C. oertzeni* Forel, *Crematogaster schmidti* (Mayr), *C. sordidula* (Nylander), *Lasius alienus* (Förster), *Plagiolepis pygmaea* (Latreille), *Temnothorax exilis* Emery, *Tetramorium kephalosi* Salata & Borowiec.

**Ionian Islands, Zakynthos, 1.2 km SE of Loucha**: *Aphaenogaster balcanica* (Emery), A. cf. epirotes, *Bothriomyrmex communista* Santschi, *Camponotus aethiops* (Latreille), *C. dalmaticus* (Nylander), *C. gestroi* Emery, *C. kiesenwetteri* (Roger), *C. lateralis* (Olivier), *Crematogaster schmidti* (Mayr), *Lepisiota frauenfeldi* (Mayr), *Messor wasmanni* Krausse, *Tapinoma erraticum* (Latreille), *Temnothorax bulgaricus* (Forel), *T. exilis* (Emery), *T. graecus* (Forel), *T. rogeri* Emery, T. cf. tergestinus, *Tetramorium diomedeum* Emery; **1.4 km S of Lithakia**: *Aphaenogaster balcanica* (Emery), *Camponotus dalmaticus* (Nylander), *C. kiesenwetteri* (Roger), *C. oertzeni* Forel, *Crematogaster schmidti* (Mayr), *C. sordidula* (Nylander), *Lepisiota frauenfeldi* (Mayr), *L. melas* (Emery), *Messor wasmanni* Krausse, Pheidole cf. pallidula, *Plagiolepis pygmaea* (Latreille), *Temnothorax rogeri* Emery, T. cf. tergestinus; **330 m S of Stimies**: *Aphaenogaster balcanica* (Emery), *Bothriomyrmex communista* Santschi, *Camponotus kiesenwetteri* (Roger), *C. oertzeni* Forel, *Crematogaster schmidti* (Mayr), *C. sordidula* (Nylander), *Messor wasmanni* Krausse, Pheidole cf. pallidula, *Plagiolepis pygmaea* (Latreille), *Temnothorax exilis* (Emery), *T. graecus* (Forel); **580 m SW of Lithakia**: Aphaenogaster cf. epirotes, *Camponotus aethiops* (Latreille), *C. dalmaticus* (Nylander), *C. gestroi* Emery, *C. kiesenwetteri* (Roger), *C. lateralis* (Olivier), *Crematogaster schmidti* (Mayr), *C. sordidula* (Nylander), *Messor ibericus* Santschi, *M. wasmanni* Krausse, *Plagiolepis pygmaea* (Latreille), *Temnothorax exilis* (Emery), T. cf. tergestinus, *Tetramorium diomedeum* Emery; **600 m E of Ag. Leon**: *Aphaenogaster balcanica* (Emery), *A. muelleriana* Wolf, *Camponotus aethiops* (Latreille), *C. dalmaticus* (Nylander), *C. kiesenwetteri* (Roger), *C. lateralis* (Olivier), *Crematogaster schmidti* (Mayr), *Lepisiota frauenfeldi* (Mayr), *L. melas* (Emery), *Messor ibericus* Santschi, *M. wasmanni* Krausse, Pheidole cf. pallidula, *Plagiolepis pygmaea* (Latreille), *Temnothorax bulgaricus* (Forel), *T. rogeri* Emery, T. cf. tergestinus, *Tetramorium kephalosi* Salata & Borowiec.

**Peloponnese, Messinia, 2 km E of Kalamata**: *Aphaenogaster balcanica* (Emery), A. cf. muelleriana, *Camponotus gestroi* Emery, *C. ionius* Emery, *C. kiesenwetteri* (Roger), *C. laconicus* Emery, *C. lateralis* (Olivier), *Crematogaster schmidti* (Mayr), *C. sordidula* (Nylander), *Formica clara* Forel, *Lasius illyricus* Zimmermann, *L. lasioides* (Emery), *L. neglectus* Van Loon, Boomsma & Andrasfalvy, *Lepisiota frauenfeldi* (Mayr), *Messor wasmanni* Krausse, *Nylanderia jaegerskioeldi* (Mayr), *Pheidole pallidula* (Nylander), *Plagiolepis pygmaea* (Latreille), *Temnothorax graecus* (Forel), T. cf. luteus, *T. recedens* (Nylander), *T. rogeri* Emery, Tetramorium cf. caespitum; **Egaleo Mts., 1.4 km S of Flesiada**: *Aphaenogaster balcanica* (Emery), A. cf. muelleriana, *Camponotus aethiops* (Latreille), *C. dalmaticus* (Nylander), *Crematogaster schmidti* (Mayr), *C. sordidula* (Nylander), *Lasius lasioides* (Emery), *Pheidole pallidula* (Nylander), *Plagiolepis pygmaea* (Latreille), *Tapinoma erraticum* (Latreille), *Temnothorax exilis* (Emery), *T. laconicus* Csősz et al., *T. morea* Csősz, Salata & Borowiec; **Kalamata, old centre**: *Aphaenogaster balcanica* (Emery), *Crematogaster schmidti* (Mayr), *Formica clara* Forel, *Lepisiota frauenfeldi* (Mayr), *L. melas* (Emery), *Messor hellenius* Agosti & Collingwood, *Pheidole pallidula* (Nylander), *Plagiolepis pygmaea* (Latreille), Solenopsis cf. lusitanica, *Temnothorax graecus* (Forel), Tetramorium cf. hungaricum, *Trichomyrmex perplexus* Radchenko; **Kalamata, railway park**: *Camponotus*lateralis (Olivier), *Crematogaster schmidti* (Mayr), *Formica clara* Forel, *Lasius illyricus* Zimmermann, *L. lasioides* (Emery), *L. neglectus* Van Loon, Boomsma & Andrasfalvy, *Messor wasmanni* Krausse, *Pheidole indica* Mayr, *Plagiolepis pygmaea* (Latreille), *Temnothorax graecus* (Forel), Tetramorium cf. caespitum; **Kondovounia Mts., 0.8 km N of Koromilea**: *Aphaenogaster balcanica* (Emery), A. cf. muelleriana, *Camponotus dalmaticus* (Nylander), *C. gestroi* Emery, *C. laconicus* Emery, *C. lateralis* (Olivier), *Cataglyphis nodus* (Brullé), *Crematogaster schmidti* (Mayr), *Lasius lasioides* (Emery), *Lepisiota frauenfeldi* (Mayr), *Pheidole pallidula* (Nylander), *Plagiolepis pygmaea* (Latreille), Temnothorax cf. bulgaricus, *T. exilis* (Emery), *T. laconicus* Csősz et al., *T. morea* Csősz, Salata & Borowiec, *T. recedens* (Nylander); **Taygetos Mts., 0.8 km W of Eleochori**: *Aphaenogaster balcanica* (Emery), *Camponotus gestroi* Emery, *C. ionius* Emery, *C. kiesenwetteri* (Roger), *Lepisiota nigra* (Dalla Torre), *Messor wasmanni* Krausse, *Pheidole pallidula* (Nylander), *Plagiolepis pygmaea* (Latreille), *Temnothorax exilis* (Emery), *Tetramorium kephalosi* Salata & Borowiec.

#### Temnothorax turcicus

Taxon classificationAnimaliaHymenopteraFormicidae

(Santschi, 1934)

90FD88EB-86E0-5E6B-A92D-B96881D883FA

**Figures 7, 8.**
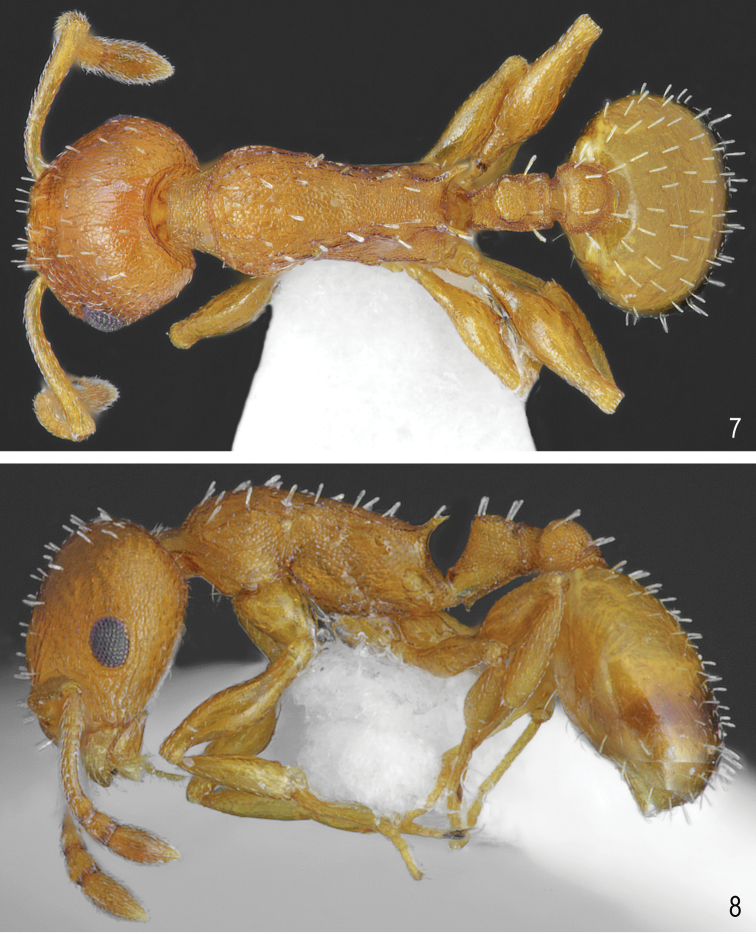
Worker of *Temnothorax turcicus* (Santschi) **7** Dorsal **8** Lateral.

Figs 7 , 8 , 11 , 15

Leptothorax turcicus Santschi, 1934: 278.

##### Type material.

**Syntype**, worker (pin): • [TURKEY]: Izmir | 29.VII.33, Santschi || Type || Sammlung | Dr. F. Santschi | Kairouan | ANTWEB | CASENT0913009 (NHMB).

##### Other material.

**Greece. North Aegean, Lesbos**: • 2w. (pin) (CASENT0846851-CASENT0846852): n. Ahladeri, 39.15958N/26.29292E, 9 m, 2015-06-10, leg. L. Borowiec. **Macedonia, Chalkidiki**: • 12w. (pin) (CASENT0846853-CASENT0846864): Holomontas, Taxiarhis vicinity, mountain deciduous forest, in leaf litter, 40.4N/23.51666E, 594 m, 2009-08-30, leg. L. Borowiec; • 2w. (pin) (CASENT0846865-CASENT0846866): Holomontas, Stagira, on wall in deciduous forest, 40.52896N/23.74872E, 539 m, 2009-09-03, leg. L. Borowiec; • 5w. (pin) (CASENT0846867-CASENT0846871): Holomontas, Stagira-Neochori road, on wall in deciduous forest, 40.51666N/23.7E, 512 m, 2009-09-03, leg. L. Borowiec. **Macedonia, Kavalas**: • 1w. (pin) (CASENT0846872): Nestos river near Komnina, 41.169N/24.6966E, 100 m, 1999-10-10, leg. E. Nikolakakis. **Macedonia, Pieria**: • 5w. (pin) (CASENT0846873-CASENT0846877), 2w. (EtOH): road to P. Poroi loc. 1, roadsides with shrubs, 39.97963N/22.61563E, 110 m, 2019-05-17, leg. L. Borowiec; • 6w. (EtOH): road to P. Poroi loc. 2, roadsides with shrubs, 39. 97627N/22.61146E, 185 m, 2019-05-17, leg. L. Borowiec; •14w. (pin) (CASENT0846878-CASENT0846891), 40w. (EtOH): road to P. Poroi loc. 3, roadsides with shrubs, 39.96863N/22.60494E, 260 m, 2019-05-17, leg. L. Borowiec. **Peloponnese, Arcadia**: • 1w. (pin) (CASENT0846892): 3.2 km NW Polidroso, 1000 m, 37.19874N/2257603E, 1000, 2016-06-18, leg. L. Borowiec. **Peloponnese, Laconia**: • 6w. (pin) (CASENT0846893-CASENT0846898): Parnon Mts., 5 km NE of Karies, 37.324N/22.538E, 1000 m, 2000-04-29, leg. A. Schulz & K. Vock (3w DBET, 3w PW). **Sterea Ellas, Euboea**: • 1w. (pin) (CASENT0846899): 1 km NE of Amfithea, 38.5519N/23.79546, 200 m, 2018-06-10, leg. L. Borowiec. **Thessaly, Larissa**: • 1w. (pin) (CASENT0846900): Kato Olimbos Mts, 6.1 km S of Kalipefki, 39.91322N/22.4641E, 855 m, 2017-05-09, leg. L. Borowiec.

##### Terra typica.

Greece, Thessaly, Mt. Ossa.

##### Differential diagnosis.

Differentiation from *T. kemali*, *T. brackoi* and *T. messiniaensis* – see differential diagnosis in *T. messiniaensis*. *Temnothorax turcicus* differs from specimens of *T. aveli* with long propodeal spines in thin dark band on first gastral tergite, head not darker from mesosoma, mesosoma less convex in profile and propodeal spines directed slightly more upwards; from *T. lagrecai* (Baroni Urbani, 1964), species described and known only from Sicily, differs in petiolar node dorsum flat or slightly convex and distinctly bigger mesosoma size - ML 0.595 ± 0.50 (0.517–0.680) vs. ML = 0.779 ± 0.05 (0.677–0.832).

##### Redescription.

Worker (n = 10): HL: 0.670 ± 0.03 (0.614–0.696); HW: 0.573 ± 0.03 (0.522–0.596); SL: 0.465 ± 0.02 (0.431–0.484); EL: 0.158 ± 0.009 (0.149–0.174); EW: 0.118 ± 0.01 (0.102–0.137); WL: 0.779 ± 0.05 (0.677–0.832); PSL: 0.192 ± 0.005 (0.186–0.199); SDL: 0.118 ± 0.007 (0.106–0.124); PEL: 0.271 ± 0.015 (0.248–0.286); PPL: 0.173 ± 0.01 (0.149–0.186); PEH: 0.206 ± 0.015 (0.186–0.230); PPH: 0.205 ± 0.02 (0.174–0.236); PNW: 0.394 ± 0.02 (0.360–0.422); PLW: 0.169 ± 0.007 (0.161–0.180); PPW: 0.219 ± 0.01 (0.199–0.230); CI: 85.6 ± 1.2 (83.6–86.8); SI1: 69.4 ± 0.6 (68.5–70.2); SI2: 81.1 ± 1.3 (79.2–82.7); MI: 50.7 ± 1.3 (49.3–53.2); EI1: 75.3 ± 8.8 (66.1–91.7); EI2: 17.7 ± 1.3 (16.4–20.0); PI: 132.3 ± 9.2 (120.0–148.4); PPI: 84.8 ± 4.5 (75.8–88.9); PSI: 162.7 ± 7.5 (155.0–176.5).

##### Colour.

Whole body uniformly yellow to dark yellow, sometimes club in darker yellow colouration. Gaster yellow, only the first gaster tergite with very thin, dark band on its posterior part (Figs 7, 8). **Head.** Oval, 1.16 times as long as wide, lateral surfaces below and above eyes gently convex, posterior edges convex, occipital margin of head straight or slightly convex (Fig. 15). Anterior margin of clypeus slightly convex, medial notch absent. Eyes moderate, oval, 1.34 times as long as wide. Antennal scape short, in lateral view slightly curved, 0.69 times as long as length of the head, in apex gradually widened, its base with small, triangular tooth, funiculus long, club 3 segmented (Figs 12, 15). Surface of scape with very fine microreticulation, shiny, covered with thin, moderate dense, decumbent setae. Mandibles rounded with thick sparse, longitudinal striae, shiny. Clypeus shiny with thick, sparse, longitudinal striae, area between striae smooth and shiny. Frontal carinae short, not extending beyond frontal lobes. Antennal fossa deep, with irregular, dense to sparse, thick rugosity and sometimes with a few thin, roundly curved striae, surface between thick sculpture smooth or with sparse microreticulation. Frontal lobes narrow, smooth with slight, dense longitudinal striation (Fig. 15). Frons, vertex and temples with dense, thick, longitudinal reticulation, central surface of frons and vertex with longitudinal reticulation sparser or reduced, with additional thin, longitudinal striation, striae sometimes interrupted, surface between striation smooth and shiny; malar area with irregular, thick, reticulation, space between reticulation smooth or with very sparse microreticulation, shiny; genae with sparser, than on frons, and thick reticulation, shiny (Fig. 15). Frons and vertex with erect, pale, short and thick setae. **Mesosoma.** Elongate, 1.98 times as long as wide, slightly arched in profile. Metanotal groove absent. Pronotum convex on sides. Propodeal spines long, directed upward, with base slightly to moderate wider than apex, tips sharp (Fig. 8). Whole surface with dense, reticulation, sometimes its dorsal surface and lateral surfaces of pronotum and mesonotum with additional thick, sparse longitudinal wrinkles. Area between thick sculpture shiny, smooth or sometimes with sparse, fine microreticulation (Fig. 8). Entire mesosoma bearing erect, pale, short and thick setae (Fig. 8). **Petiole.** In lateral view, with short peduncle, node moderate high, with anterior face straight, and posterior face convex and dorsum flat or slightly convex. Peduncle and petiolar node shiny, with thick, dense reticulation, area between rugae smooth, dorsum with sparser reticulation. Dorsal surface bearing sparse, short, erect setae (Fig. 8). **Postpetiole.** In lateral view, regularly convex, apical half with gently convex sides (Fig. 8), on the whole surface shiny, with thick, dense reticulation, dorsum with sparser reticulation; area between rugae smooth. Dorsal surface bearing sparse, short, erect setae. **Gaster.** Gaster smooth and shiny, bearing erect, thin, pale setae (Figs 7, 8).

**Figures 9–12.**
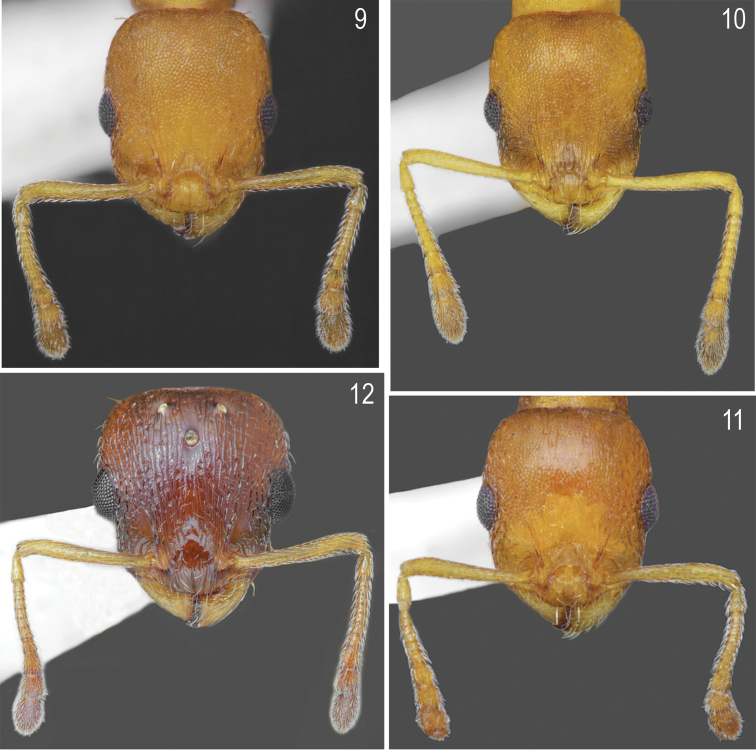
Head and antennae **9** Worker of *Temnothorax brackoi* sp. nov. **10** Worker of *Temnothorax messiniaensis* sp. nov. **11** Worker of *Temnothorax turcicus* (Santschi) **12** Gyne of *Temnothorax messiniaensis* sp. nov.

##### General distribution.

Eastern Austria, Bulgaria, Croatia, Greece: Macedonia, North Aegean Islands, Sterea Ellas, Peloponnese, and Thessaly, Hungary, Slovakia, western Turkey.

##### Comment.

We examined a syntype of *Temnothorax tauricus* (Ruzsky, 1902) preserved in Forel’s collection (MHNG) and it appears to be very similar to specimens of *T. turcicus* collected in Greece. The only difference is a slightly darkened antennal club in the syntype specimen of *T. tauricus* (all studied specimens of *T. turcicus* have antennae uniformly yellow). We discussed this issue with Alex Radchenko (Kiev, Ukraine) who confirmed that all 17 syntypes of *T. tauricus* preserved in Karavaiev’s collection (Kiev, Ukraine) also have slightly darkened antennal club. In our opinion this difference could be an infraspecific variation. Within nest samples of *Temnothorax messiniaensis*, a member of the *aveli* species group, we observed single specimens with more or less darkened antennal club. *Temnothorax tauricus* was recorded from Caucasus and southern Ukraine but is sympatric with *T. turcicus* in Bulgaria and Greece. *Temnothorax tauricus* have nests in dry stems of herbs, grasses or rarely in soil under stones (Radchenko 2016) and by those preferences reminds species of the *T. aveli* species group. Clarification of taxonomic relation between those two taxa requires further study based on material collected from the whole distribution range of both species. If our supposition on the conspecifity of both taxa is confirmed, then the name *T. tauricus* will have priority over the name *T. turcicus*.

##### Biology.

Specimens collected on shadow localities, from seacoast to 1000 m a.s.l. Foraging workers were observed on herbs in stream valley of tourist resort, valleys with *Platanus* trees, mountain coniferous forest and mountain pastures close to border of coniferous forest. Nests were not found, probably like other species of this group, are located inside dry stems of herbs.

The following ant species were recorded in the same areas as *T. turcicus*:

For localities on **Macedonia**: Pieria, road to P. Poroi loc. 1, Pieria, road to P. Poroi loc. 2, Pieria, road to P. Poroi loc. 2, and **Thessaly, Larissa, Mt. Ossa, Kokkino Nero**: see *Temnothorax brackoi*.

**North Aegean, Lesbos, near Ahladeri**: *Camponotus dalmaticus* (Nylander), *C. lateralis* (Olivier), *C. sanctus*, *Crematogaster ionia* Forel, *Lasius neglectus* Van Loon, Boomsma & Andrasfalvy, *Monomorium monomorium* Bolton, *Pheidole pallidula* (Nylander), *Plagiolepis perperamus* Salata et al., *Temnothorax bulgaricus* (Forel), T. cf. luteus, *Tetramorium rhodium* Emery.

**Peloponnese, Arcadia, 3.2 km NW Polidroso**: Aphaenogaster cf. subterranea, *Camponotus aethiops* (Latreille), *C. dalmaticus* (Nylander), *C. nitidescens* Forel, *C. vagus* (Scopoli), *Cataglyphis nodus* (Brullé), *Crematogaster ionia* Forel, *Formica cunicularia* Latreille, *F. fusca* Linnaeus, *Lasius bomycina* Seifert & Galkowski, *L. flavus* (Fabricius), *L. illyricus* Zimmermann, Pheidole cf. pallidula, *Plagiolepis pygmaea* (Latreille), *Temnothorax crasecundus* Seifert & Csősz, *T. helenae* Csősz et al., *T. laconicus* Csősz et al., T. cf. unifasciatus, Tetramorium cf. caespitum.

**Sterea Ellas, Euboea, 1 km NE of Amfithea**: *Camponotus lateralis* (Olivier), *Cataglyphis nodus* (Brullé), *Crematogaster schmidti* (Mayr), Pheidole cf. pallidula, *Plagiolepis pygmaea* (Latreille), *Temnothorax bulgaricus* (Forel), *T. recedens* (Nylander).

**Thessaly, Larissa, Kato Olimbos Mts, 6.1 km S of Kalipefki**: Aphaenogaster*epirotes* (Emery), *Bothriomyrmex communista* Santschi, *Camponotus aethiops* (Latreille), *C. oertzeni* Forel, *C. piceus* (Leach), *Formica cunicularia* Latreille, *Lasius alienus* Förster, *Messor mcarthuri* Steiner et al., *M. wasmanni* Krausse, *Plagiolepis pygmaea* (Latreille), Temnothorax cf. unifasciatus, Tetramorium cf. caespitum.

### A key to Greek members of the *T. aveli* species group

**Table:** 

1	Propodeal spines short to moderate (PSI<155), triangular, with wide base (Fig. 2); head on whole frontal surface with regular reticulation, without longitudinal striation, with dull background (Fig. 13)	***T. brackoi* sp. nov.**
–	Propodeal spines long (PSI > 155), thin, with base slightly to moderate wider than base (Figs 4, 8); head regularly reticulate but often with additional thin, longitudinal striation and partly shiny background (Figs 14, 15)	**2**
2	Central surface of frons with dense, thick, longitudinal reticulation and sometimes with additional thin, longitudinal striation (Fig. 10); first gaster tergite with wide, dark band on its posterior part (Figs 3–4); gena, mid and hind femora often obscured in middle	***T. messiniaensis* sp. nov.**
–	Central surface of frons and vertex with longitudinal reticulation sparser than of rest of head or reduced (Fig. 12); first gaster tergite with very thin, dark band on its posterior part (Figs 7, 8); gena, mid and hind femora never obscured in middle	***T. turcicus* (Santschi)**

**Figures 13–15.**
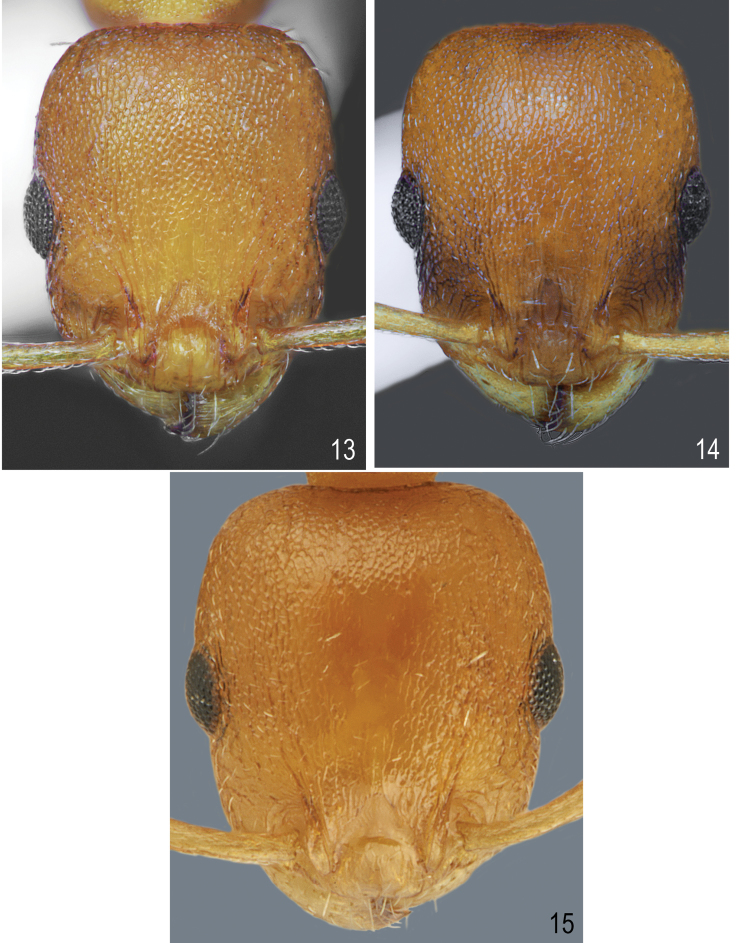
Head sculpture **13** Worker of *Temnothorax brackoi* sp. nov. **14** Worker of *Temnothorax messiniaensis* sp. nov. **15** Worker of *Temnothorax turcicus* (Santschi).

### Description of a new species of the *Temnothorax nylanderi* species group

#### Temnothorax triangularis sp. nov.

Taxon classificationAnimaliaHymenopteraFormicidae

B8DBB77A-FD37-5F93-AFAE-0F4064C150BD

http://zoobank.org/4D263567-874F-45D1-870C-E64C9750A9F5

Figs 16–17 , 18–20 , 21, 22 , 23–24

##### Differential diagnosis.

*Temnothorax triangularis* belongs to the *T. nylanderi* species group. It differs from most of members of this group in uniformly brown body with darker frons and posterior band of first gastral tergite, and absent or very shallow, inconspicuous metanotal groove. There are four other known species with dark body colouration: *T. laconicus*, *T. artvinensis*, *T. sordidulus*, and *T. tergestinus*. *Temnothorax triangularis* differs from all of them in extremely shallow metanotal groove and frons lacking reticulation and covered with dense, thick, longitudinal striations, additionally frons centre has sculpture weaker or reduced, it differs also from *T. laconicus* and *T. artvinensis* in shorter, triangular propodeal spines.

##### Etymology.

Named after short, triangular propodeal spines.

##### Type material.

**Holotype**, worker (pin) (CASENT0846901): GREECE, Sterea Ellas, Eubea | 2.4 km SW of Stropones | 38,60327N/23,87E, 1025 m |10 VI 2018, L. Borowiec || Collection L. Borowiec | Formicidae | LBC-GR02682 (MNHW).

**Paratypes**, 25w., 1Q.(pin) (CASENT0846661-CASENT0846691): the same nest sample as holotype (DBET, BMNH, CASC, MHNG); 5w. (EtOH): the same locality as holotype, collected in litter (DBET); 11w, 1Q (pin) (CASENT0846692-CASENT0846704): GREECE, Sterea Ellas | Eubea |3.7 km SW of Metochi, 830 m | 38.60402N/23.91683E, | 13 VI 2018, L. Borowiec (DBET).

##### Terra typica.

Euboea, Greece.

##### Other material.

**GREECE, Sterea Ellas, Euboea**: 2w. (EtOH): 1.5 km SW of Koutourla, 38.62838N/23.92772E, 695 m, 2018-06-13, leg. L. Borowiec; 1w.(pin) (CASENT0846902), 2w. (EtOH): 2.3 km S of Stropones, 38.9933N/23.87807E, 860 m, 2018-06-10, leg. L. Borowiec; 2w. (EtOH): 2.7 km SE of Stropones, 38.59851N/23.9085E, 855 m, 2018-06-13, leg. L. Borowiec; 3w. (EtOH): 2.9 km S of Stropones, 38.59133N/23.88562E, 880 m, 2018-06-13, leg. L. Borowiec; 43w. (EtOH): 3.7 km SW of Metochi, 38.60402N/23.91683E, 830 m, 2018-06-13, leg. L. Borowiec.

**Description of worker** (n = 10): HL: 0.684 ± 0.01 (0.671–0.708); HW: 0.603 ± 0.02 (0.578–0.650); SL: 0.504 ± 0.02 (0.484–0.534); EL: 0.140 ± 0.01 (0.124–0.149); EW: 0.105 ± 0.006 (0.093–0.112); WL: 0.769 ± 0.02 (0.742–0.820); PSL: 0.161 ± 0.02 (0.143–0.183); SDL: 0.119 ± 0.006 (0.112–0.130); PEL: 0.294 ± 0.02 (0.273–0.323); PPL: 0.182 ± 0.008 (0.174–0.199); PEH: 0.245 ± 0.01 (0.236–0.270); PPH: 0.231 ± 0.01 (0.217–0.248); PNW: 0.403 ± 0.01 (0.388–0.435); PLW: 0.177 ± 0.009 (0.168–0.199); PPW: 0.241 ± 0.01 (0.230–0.267); CI: 88.2 ± 2.2 (85.3–92.7); SI1: 73.7 ± 1.5 (71.2–76.4); SI2: 83.6 ± 2.4 (78.9–87.5); MI: 52.4 ± 1.2 (50.8–54.7); EI1: 75.0 ± 4.2 (68.8–85.0); EI2: 15.3 ± 1.0 (13.6–16.7); PI: 120.1 ± 5.4 (108.0–128.8); PPI: 79.0 ± 2.8 (75.0–85.7); PSI: 135.8 ± 12.4 (120.0–155.6).

##### Colour.

**Figures 16, 17.**
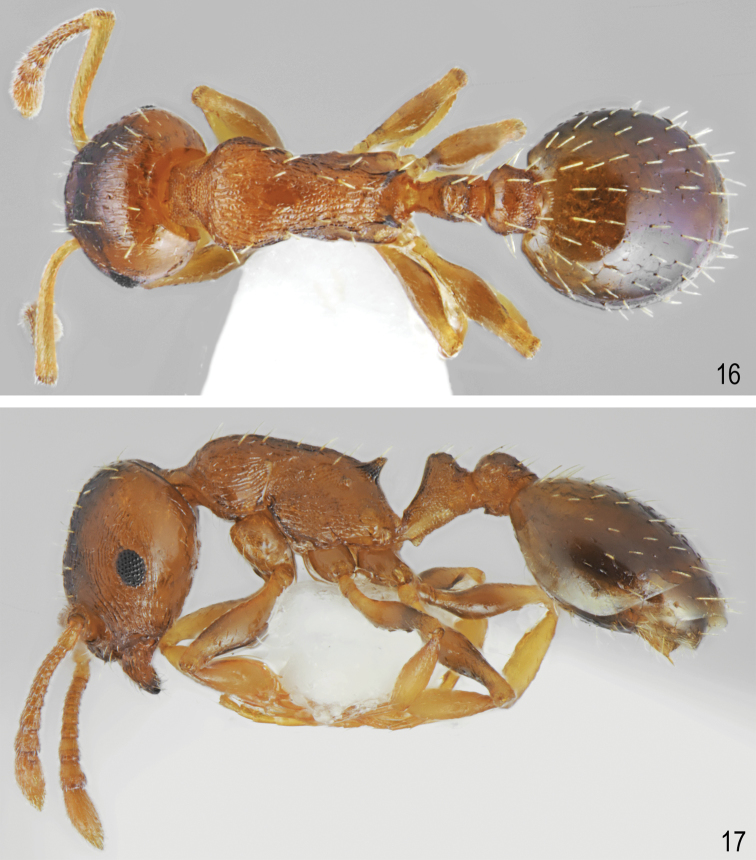
Worker of *Temnothorax triangularis* sp. nov. **16** Dorsal **17** Lateral.

**Figures 18–20.**
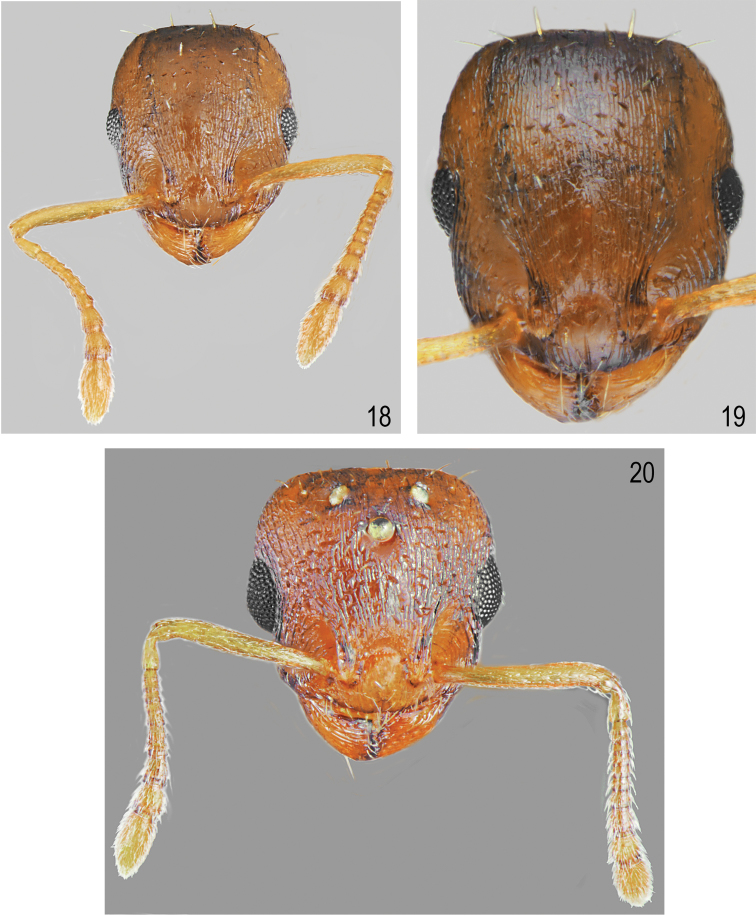
*Temnothorax triangularis* sp. nov. **18** Worker head and antennae **19** Worker head sculpture **20** Gyne head and antennae.

**Figures 21, 22.**
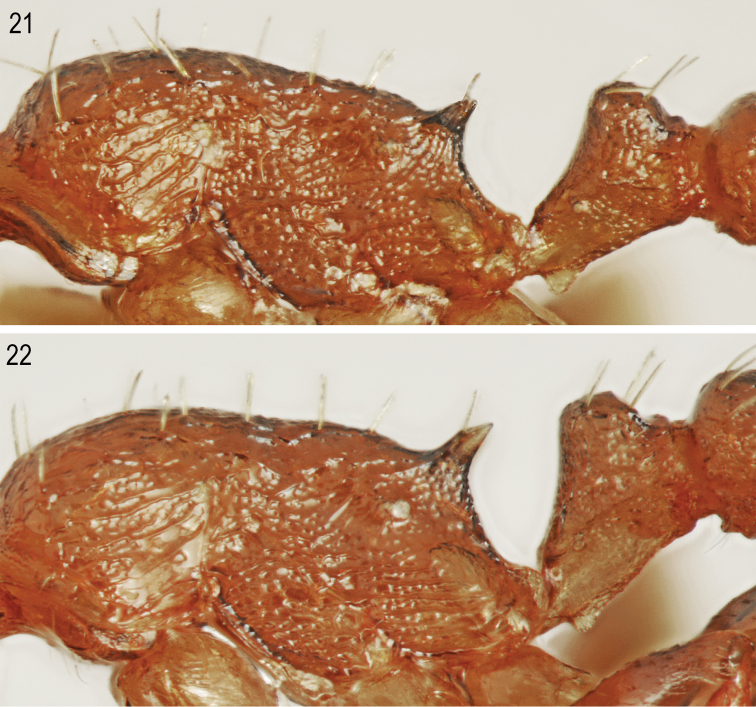
Variation of propodeal spines of *Temnothorax triangularis* sp. nov.

Whole body uniformly brown to bright brown, sometimes mesosoma and genae brighter. Legs and antennae bright brown to dark yellow, femora in central part darkened (Figs 16, 17). **Head.** Oval, 1.14 times as long as wide, lateral surfaces below and above eyes gently convex, posterior edges convex, occipital margin of head straight or slightly concave (Figs 18, 19). Anterior margin of the clypeus slightly convex, medial notch absent. Eyes small, oval, 1.33 times as long as wide. Antennal scape short, in lateral view slightly curved, 0.73 times as long as length of the head, in apex gradually widened, its base with small, triangular tooth, funiculus long, club 3-segmented (Fig. 18). Surface of scape with very fine microreticulation, shiny, covered with thin, moderate dense, decumbent setae. Mandibles rounded with thick sparse, longitudinal striae, shiny. Clypeus shiny with thick, longitudinal striae, area between striae shiny with few longitudinal wrinkles. Frontal carinae short, not extending beyond frontal lobes. Antennal fossa deep, with sparse, thin roundly curved striae, area between striae with sparse and fine reticulation, shiny. Frontal lobes narrow, smooth with slight, dense longitudinal striation (Figs 18, 19). Space between frontal carinae and vertex with dense, thick, longitudinal striation, sparser or reduced in the central part, striae sometimes interrupted, surface between striae smooth and shiny; space between frontal carinae ad eyes, temples and malar area with longitudinal, thick reticulation, space between reticulation smooth or with very sparse microreticulation, shiny; genae with very sparse thick reticulation, partly smooth, always shiny. Frons and vertex with erect, pale, short and thick setae. **Mesosoma.** Elongate, 1.9 times as long as wide, slightly arched in profile. Metanotal groove absent or very shallow, inconspicuous. Pronotum convex on sides. Propodeal spines short, triangular, with wide base, directed upward, with angulate tips (Fig. 21), only in fewer than 20% of specimens are propodeal spines moderately long, with wide bases and sharp tips (Fig. 22). Lateral surfaces of pronotum with thick and sparse longitudinal striation or reticulation, its dorsal surface with thick, sparse, irregular reticulation; mesonotum and propodeum on the whole surface with thick, denser than on pronotum, irregular reticulation. Area between thick sculpture shiny, smooth or sometimes with sparse, fine microreticulation (Fig. 17). Entire mesosoma bearing erect, pale, short and thick setae (Fig. 17). **Petiole.** In lateral view, with short peduncle, node high, with anterior face flat to slightly convex, and posterior face and dorsum convex. Peduncle and petiolar node shiny, with thick, dense reticulation, area between rugae smooth, dorsum with sparser reticulation. Dorsal surface bearing sparse, short, erect setae (Figs 21, 22). **Postpetiole.** In lateral view, regularly convex, apical half with gently convex sides (Figs 21, 22), on the whole surface shiny, with thick, dense reticulation, dorsum with sparser reticulation; area between rugae smooth. Dorsal surface bearing sparse, short, semierect to erect setae. **Gaster.** Smooth and shiny, bearing erect, thin, pale setae (Figs 16, 17).

**Figures 23, 24.**
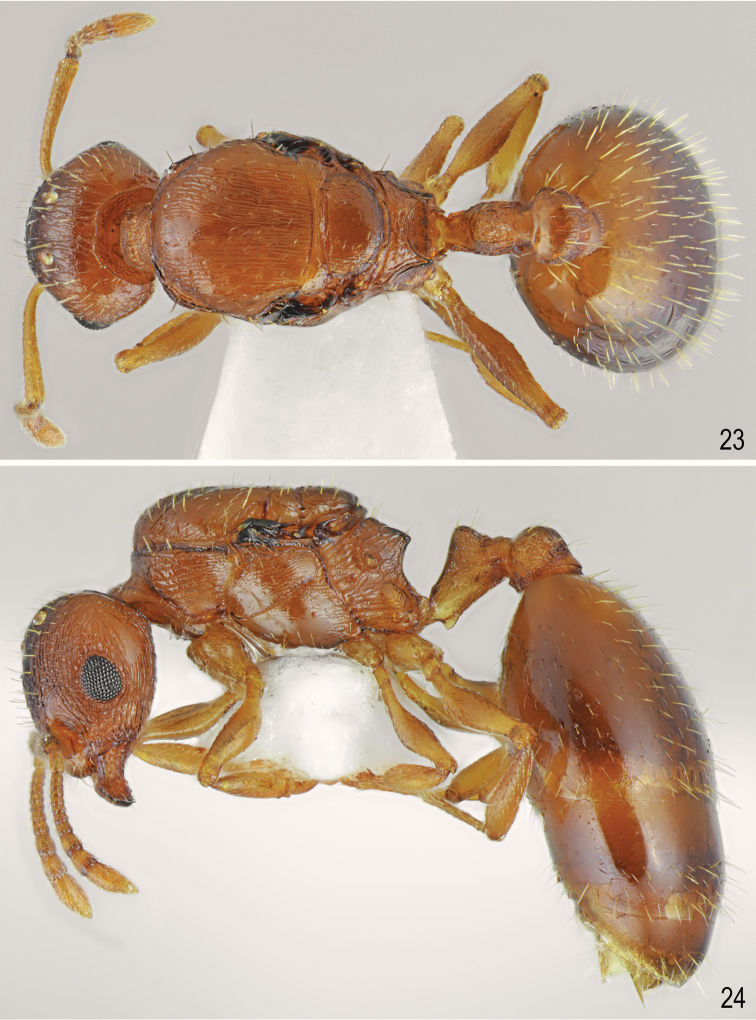
Gyne of *Temnothorax triangularis* sp. nov. **23** Dorsal **24** Lateral.

**Description of gyne** (n = 2). **Colour.** Head brown, temples slightly brighter coloured than frontal parts. Antennae uniformly yellow. Mesosoma, petiole and postpetiole bright brown, legs yellow. First gastral tergite mostly dark brown with yellowish brown spot basally, remaining tergites brown with dark brown posterior margins (Figs 23, 24). **Head.** Eyes big, almost round [EL / HW: 0.26]. Antennal scape short [SL / HW: 0.76], not reaching occipital margin of head. Clypeus smooth and shiny laterally with diffused, longitudinal carinulae. Antennal fossa deep, rugulose with concentric carinae. Frontal lobes wide 0.43 times as wide as head width, rugulose with thick longitudinal costae, interstices microreticulate (Fig. 20). Frons shiny, entire surface longitudinally costate and rugose, interstices distinctly microreticulate. Area above eyes and sides of head rugulose and partly longitudinally costate, only small area behind eyes smooth and shiny. Entire head bearing erect, pale and thin setae. **Mesosoma.** Pronotum with thick rugosities in anterior part, sides with thick rugosity and dense longitudinal costae. Surface between rugosities microreticulate. Scutum with dense, thick longitudinal costae and microreticulation between costae but appears shiny. Scutellum laterally with thick longitudinal costae, to the centre costae partly diffused, along middle smooth and shiny area (Figs 23, 24). Metanotum with fine longitudinal costae and microreticulated background. Propodeum with area above propodeal spines with transverse and on sides with longitudinal costae and microreticulated background. Propodeal spines short [PSL / HW: 0.28], triangular, with wide base, straight and angulate apex. Area between and below propodeal spines with distinct microreticulation tends to form transverse ridges. Anepisternum and katepisternum with gentle, dense longitudinal costae. Metaepisternum and metakatepisternum, with dense, longitudinal costae and shiny area close to ventral margin. Surface between costae microreticulate. Dorsal surface of mesosoma with sparse, erect, long, thick and pale setae (Figs 23, 24). **Petiole and postpetiole.** Microreticulate, the entire surface punctate to rugulose, dorsal surface longitudinally costulate. **Gaster.** Smooth and shiny, bearing sparse, long, erect setae (Figs 23, 24).

##### General distribution.

Greece, Sterea Ellas, Euboea.

##### Biology.

Alpine species. Ants were observed on stones, dry branches, and herbs in coniferous forest, or coniferous forest with an admixture of chestnut. Nests were found in the dry branches of conifers lying on the ground.

The following ant species were recorded in the same areas as *T. triangularis*:

**Sterea Ellas, Euboea, 1.5 km SW of Koutourla**: *Aphaenogaster subterranea* (Latreille), *Formica fusca* Linnaeus, *Lasius alienus* Förster, *L. brunneus* (Latreille), *L. illyricus* Zimmermann, *Pheidole pallidula* (Nylander), *Plagiolepis pygmaea* (Latreille), *P. pallescens* Forel, *Temnothorax helenae* Csősz et al., *T. lichtensteini* (Bondroit), *T. unifasciatus* (Latreille); **2.3 km S of Stropones**: see *Temnothorax brackoi*; **2.4 km SW of Stropones**: *Camponotus aethiops* (Latreille), *Formica fusca* Linnaeus, *F. sanguinea* Latreille, *Lasius alienus* Förster, *L. illyricus* Zimmermann, *Messor structor* (Latreille), *Temnothorax crasecundus* Seifert & Csősz, *T. unifasciatus* (Latreille), *Tetramorium impurum* (Förster); **2.7 km SE of Stropones**: *Aphaenogaster subterranea* (Latreille), A. cf. subterranea, *Camponotus aethiops* (Latreille), *C. oertzeni* Forel, *Formica fusca* Linnaeus, *Lasius alienus* Förster, *L. distinguendus* (Emery), *Tapinoma erraticum* (Latreille), *Temnothorax helenae* Csősz et al., *T. unifasciatus* (Latreille);

**2.9 km S of Stropones**: *Camponotus piceus* (Leach), *C. vagus* (Scopoli), *Lasius brunneus* (Latreille), *L. flavus* (Fabricius), *Myrmica scabrinodis* Nylander, *Temnothorax helenae* Csősz et al., *T. unifasciatus* (Latreille); **3.7 km SW of Metochi**: *Aphaenogaster subterranea* (Latreille), A. cf. subterranea, *Camponotus fallax* (Nylander), *C. vagus* (Scopoli), *Lasius illyricus* Zimmermann, *Temnothorax crasecundus* Seifert & Csősz, *T. helenae* Csősz et al., *T. unifasciatus* (Latreille).

##### Note.

Although this species has inconspicuous metanotal groove, which tends to disappear in some specimens, we decided to place it in the *Temnothorax nylanderi* group. General body shape, structure of petiole, unicolourous antennae, head and mesosoma sculpture presented by *T. triangularis* are very similar to those observed in large species of the group i.e. *T. nylanderi* (Förster), *T. crassispinus* (Karavaiev) or *T. crasecundus* Seifert & Csősz. Moreover, an inconspicuous metanotal groove was observed also in some samples of small species of the *nylanderi* group, such as *T. helenae* Csősz, Heinze & Mikó. Usually the depth of metanotal groove is more or less constant within nest samples.

**Figures 25–27.**
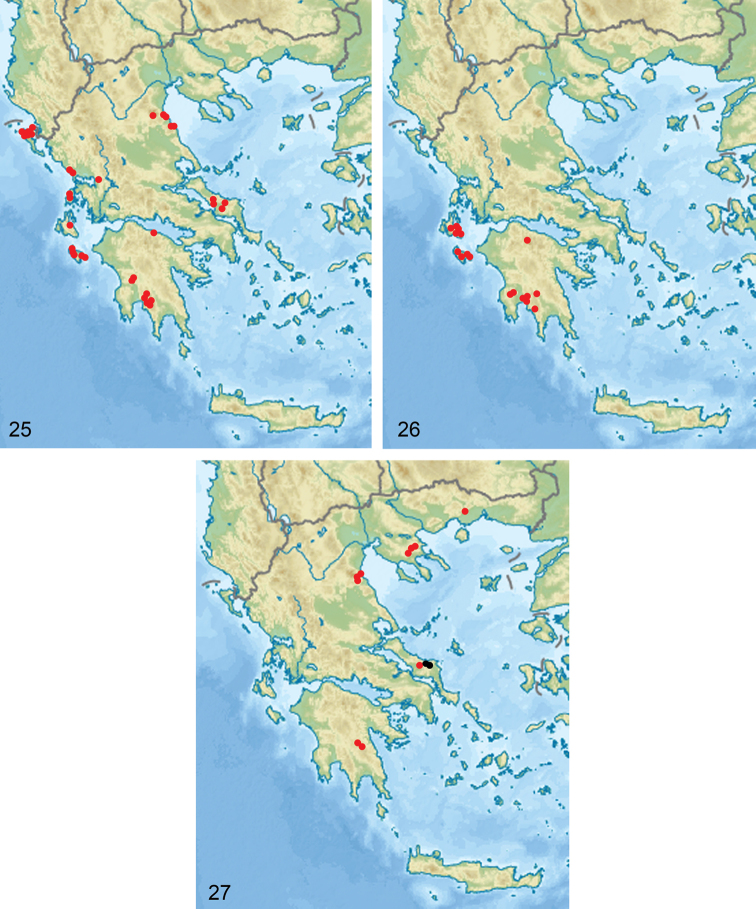
Distribution in Greece **25***Temnothorax brackoi* sp. nov. **26***Temnothorax messiniaensis* sp. nov. **27***T. turcicus* (Santschi) denoted by red circles and *T. triangularis* sp. nov. by black circles.

## Supplementary Material

XML Treatment for Temnothorax brackoi

XML Treatment for Temnothorax messiniaensis

XML Treatment for Temnothorax turcicus

XML Treatment for Temnothorax triangularis

## Footnotes

Crete, as an island, creates a well-defined unit and studies performed on Temnothorax known from the island were published in a separate paper (Salata et al. 2018). The majority of endemic taxa known from the island create a single-species group and the material collected from other Greek islands and regions adjacent to Crete confirms their uniqueness. Therefore, Cretan endemic species of Temnothorax are not included in the key.

Some specimens of the T. tuberum species group and T. interruptus species group can have very indistinct metanotal grooves. Usually it applies to single specimens within a nest sample.

## References

[B1] Bernard F (1967[1968]) Faune de l’Europe et du Bassin Méditerranéen. 3. Les fourmis (HymenopteraFormicidae) d’Europe occidentale et septentrionale. Masson, Paris, 411 pp.

[B2] Bolton B (2019) An online catalog of the ants of the world. Available from http://antcat.org [accessed (2018-08-31)]

[B3] Borowiec L (2014) Catalogue of ants of Europe, the Mediterranean Basin and adjacent regions (Hymenoptera: Formicidae). Genus 25: 1–340.

[B4] Borowiec L Salata S (2012) Ants of Greece – checklist, comments and new faunistic data (Hymenoptera: Formicidae). Genus 23: 461–563.

[B5] Borowiec L Salata S (2013) Ants of Greece – additions and corrections (Hymenoptera: Formicidae). Genus 24: 335–401.

[B6] Borowiec L Salata S (2017a) New records of ants (Hymenoptera: Formicidae) from Sterea Ellas, Greece. Acta Entomologica Silesiana 25 (online 020): 1–3. 10.5281/zenodo.834219

[B7] Borowiec L Salata S (2017b) Ants of the Peloponnese, Greece (Hymenoptera: Formicidae) Polish Journal of Entomology 86: 193–235. 10.1515/pjen-2017-0013

[B8] Borowiec L Salata S (2018a) New records of ants (Hymenoptera: Formicidae) from Epirus, Greece. Acta Entomologica Silesiana 26 (online 001): 1–22. 10.5281/zenodo.1169150

[B9] Borowiec L Salata S (2018b) Ants from Thessaly, Greece (Hymenoptera: Formicidae). Polish Journal of Entomology 87(3): 217–248. 10.2478/pjen-2018-0016

[B10] Borowiec L Salata S (2018c) Notes on ants (Hymenoptera: Formicidae) of Samos Island, Greece. Annals of the Upper Silesian Museum Bytom Entomology 27 (online 003): 1–13. 10.5281/zenodo.1481802

[B11] Borowiec L Salata S (2018d) Notes on ants (Hymenoptera: Formicidae) of Zakynthos Island, Greece. Annals of the Upper Silesian Museum Bytom Entomology 27 (online 004): 1–13. 10.5281/zenodo.1481794

[B12] Borowiec L Salata S (2018e) Notes on ants (Hymenoptera: Formicidae) of the Euboea Island, Central Greece. Annals of the Upper Silesian Museum Bytom Entomology 27 (online 005): 1–15. 10.5281/zenodo.1485235

[B13] Cagniant H Espadaler X (1997) Les *Leptothorax*, *Epimyrma* et *Chalepoxenus* du Maroc (Hymenoptera: Formicidae). Clé et catalogue des espèces. Annales de la Société entomologique de France 33: 259–84.

[B14] Csősz S Heinze J Mikó I (2015) Taxonomic Synopsis of the Ponto-Mediterranean Ants of *Temnothorax nylanderi* Species-Group. PLoS ONE 10(11): e0140000. 10.1371/journal.pone.0140000 PMC4633182 26536033

[B15] Csősz S Salata S Borowiec L (2018) Three Turano-European species of the *Temnothorax interruptus* group (Hymenoptera: Formicidae) demonstrated by quantitative morphology. Myrmecological News 26: 101–119.

[B16] Catarineu C Barberá GG Reyes-López JL (2017) A New Ant Species, *Temnothorax ansei* sp. n. (Hymenoptera: Formicidae) from the Arid Environments of South-eastern Spain. Sociobiology 64: 138–145. 10.13102/sociobiology.v64i2.1274

[B17] Galkowski C Cagniant H (2017) Contribution à la connaissance des fourmis du groupe *angustulus* dans le genre *Temnothorax* (Hymenoptera, Formicidae). Revue de l’Association Roussillonnaise d’Entomologie 26(4): 180–191.

[B18] Galkowski C Lebas C (2016) *Temnothorax conatensis* nov. sp., décrite des Pyrénées-Orientales (France) (Hymenoptera, Formicidae). Revue de l’Association Roussillonnaise d’Entomologie 25: 80–87.

[B19] Hölldobler B Wilson EO (1990) The ants. Harvard University Press, Cambridge, Mass, xii + 732 pp. 10.1007/978-3-662-10306-7

[B20] Prebus M (2017) Insights into the evolution, biogeography and natural history of the acorn ants, genus *Temnothorax* Mayr (Hymenoptera: Formicidae). BMC Evolutionary Biology 171:250. 10.1186/s12862-017-1095-8 PMC5729518 29237395

[B21] Radchenko AG (1995a) A review of the ant genus *Leptothorax* (Hymenoptera, Formicidae) of the central and eastern Palearctic. Communication 1. Subdivision into groups. Groups *acervorum* and *bulgaricus*. [In Russian.]. Vestnik Zoologii 1994(6): 22–28.

[B22] Radchenko AG (1995b) A review of the ant genus *Leptothorax* (Hymenoptera, Formicidae) of the central and eastern Palearctic. Communication 2. Groups *tuberum*, *corticalis*, *affinis*, *clypeatus*, *alinae* and *singularis*. [In Russian.]. Vestnik Zoologii 1995(2–3): 14–21.

[B23] Radchenko AG (1995c) A review of the ant genus *Leptothorax* (Hymenoptera, Formicidae) of the central and eastern Palearctic. Communication 3. Groups *nylanderi*, *korbi*, *nassonovi*, and *susamyri*. [In Russian.]. Vestnik Zoologii 1995(4): 3–11.

[B24] Radchenko AG (2016) Ants (Hymenoptera, Formicidae) of Ukraine. National Academy of Sciences of Ukraine. Kiev: I. I. Schmalhausen Institute of Zoology, 496 pp.

[B25] Radchenko AG Yusupov Z Fedoseeva EB (2015) Taxonomic notes for some Caucasian *Temnothorax* Mayr, 1861 species, with descriptions of three new species. Caucasian Entomological Bulletin 11: 161–167. 10.23885/1814-3326-2015-11-1-161-167

[B26] Salata S Borowiec L (2015) Redescription of *Temnothorax antigoni* (Forel, 1911) and description of its new social parasite *Temnothorax curtisetosus* sp. n. from Turkey (Hymenoptera, Formicidae). ZooKeys 523: 129–148. 10.3897/zookeys.523.6103 PMC4602300 26478702

[B27] Salata S Borowiec L (2018) Taxonomic and faunistic notes on Greek ants (Hymenoptera: Formicidae). Annals of the Upper Silesian Museum Bytom Entomology 27 (online 008): 1–51. 10.5281/zenodo.2199191

[B28] Salata S Borowiec L Trichas A (2018) Taxonomic revision of the Cretan fauna of the genus *Temnothorax* Mayr, 1861 (Hymenoptera: Formicidae), with notes on the endemism of ant fauna of Crete. Annales Zoologici (Warsaw) 68(4): 769–808. 10.3161/00034541ANZ2018.68.4.004

[B29] Seifert B Buschinger A Aldawood A Antonova V Bharti H Borowiec L Dekoninck W Dubovikoff D Espadaler X Flegr J Georgiadis C Heinze J Neumeyer R Ødegaard F Oettler J Radchenko A Schultz R Sharaf M Trager J Vesnic A Wiezik M Zettel H (2016) Banning paraphylies and executing Linnaean taxonomy is discordant and reduces the evolutionary and semantic information content of biological nomenclature. Insectes Sociaux 63(2): 237–242. 10.1007/s00040-016-0467-1

[B30] Sharaf MR Akbar SA Al Dhafer HM Gharbawy A Aldawood SA (2017) Taxonomy of the Myrmicine ant genus *Temnothorax* Mayr, 1861 (Formicidae: Myrmicinae) in the Arabian Peninsula. European Journal of Taxonomy 280: 1–17. 10.5852/ejt.2017.280

[B31] Vigna Taglianti A Audisio PA Biondi M Bologna MA Carpaneto GM De Biase A Fattorini S Piattella E Sindaco R Venchi A Zapparoli M (1999) A proposal for achorotype classification of the Near East fauna, in the framework of the Western Palaearctic region. Biogeographia 20: 31–59. 10.21426/B6110172

[B32] Ward PS Brady SG Fisher BL Schultz TR (2016) Phylogenetic classifications are informative, stable, and pragmatic: the case for monophyletic taxa. Insectes Sociaux 63: 489–492. 10.1007/s00040-016-0516-9 27773940 PMC5052292

